# Nonglycosidic *C–O* bond formation catalyzed by a bifunctional pseudoglycosyltransferase ValL

**DOI:** 10.1016/j.synbio.2025.04.007

**Published:** 2025-04-17

**Authors:** Ziyue Guo, Xin Zhang, Lin Zhou, Qungang Huang, Qianjin Kang, Linquan Bai

**Affiliations:** a State Key Laboratory of Microbial Metabolism, School of Life Sciences and Biotechnology, Shanghai Jiao Tong University, Shanghai, 200240, China; b College of Life Science and Technology, Tarim University, Alar, 843300, Xinjiang, China

**Keywords:** C_7_N antibiotics, Validamycin A, Pseudoglycosyltransferase, Nonglycosidic *C*–*O* bond

## Abstract

The C_7_N antibiotic validamycin A is an antifungal agent widely used as a crop protectant. It comprises a validoxylamine A unit linked to a glucose moiety, which is formed through a nonglycosidic *C–N* bond connecting a valienol moiety and a validamine moiety, a reaction catalyzed by the pseudoglycosyltransferase ValL. In this study, we analyzed the chemical composition of validamycins in *Streptomyces hygroscopicus* var. *jinggangensis* TL01. A series of novel oxygen-bridged analogues, namely, validenomycin, validomycin, and 1,1′-bis-valienol, were identified in the culture supernatants, and their chemical structures were elucidated using a combination of one- and two-dimensional nuclear magnetic resonance and mass spectrometry. Gene disruption and complementation experiments revealed that *valL* is essential for the biosynthesis of these new oxygen-bridged analogues of validamycins. Biochemical assays further demonstrated that ValL catalyzed the *C–O* bond formation between GDP-valienol and valienol-7-phosphate, producing 1,1′-bis-valienol-7-phosphate, which was subsequently dephosphorylated by ValO and glycosylated by ValG to yield validenomycin. Collectively, our findings revealed the unique ability of ValL to catalyze nonglycosidic *C–O* coupling, potentially enabling the generation of various chemical scaffolds for C_7_N family antibiotics.

## 1 Introduction

**Fig. 1:**
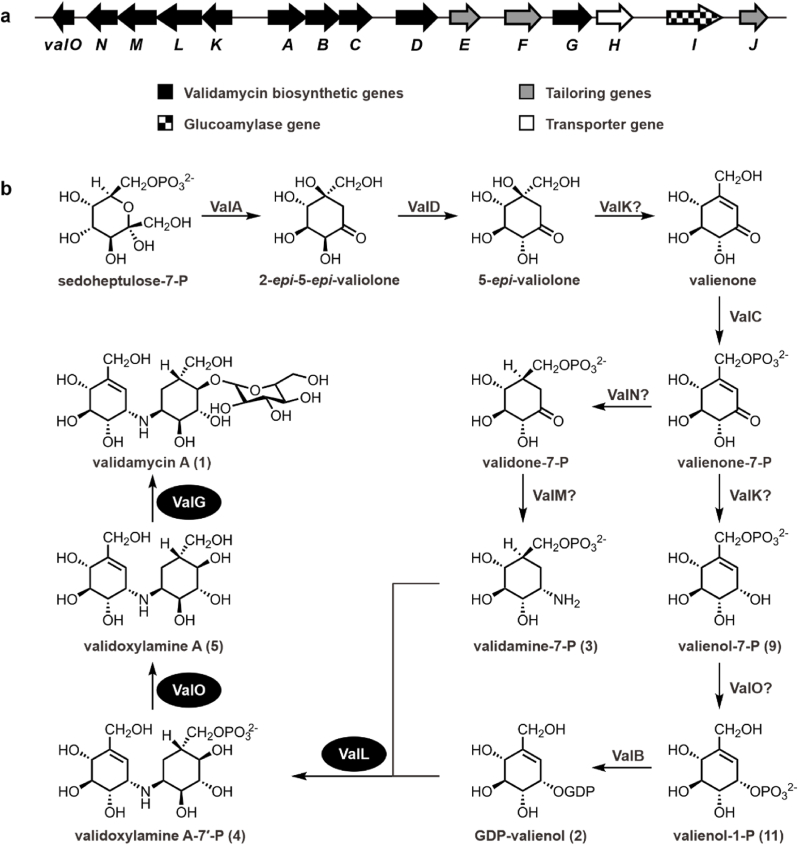
**Biosynthesis of validamycin A (1) in *S. hygroscopicus* var. *jinggangensis* TL01. a**, Validamycin A (**1**) biosynthetic gene cluster from *S. hygroscopicus* var. *jinggangensis* TL01. **b**, Proposed validamycin A (**1**) biosynthetic pathway. Enzymes highlighted with black ovals are those discussed in detail in the Results section. Question marks indicate steps in the pathway where the responsible enzyme has not been definitively identified.

C_7_N aminocyclitols, including the α-glucosidase inhibitor acarbose, the antitumor agent cetoniacytone A, and the trehalase inhibitors validamycin A (**1**) and salbostatin, have widespread applications in both medical and agricultural fields [[1], [2], [3]]. Validamycin A (**1**) is an important antifungal antibiotic extensively used to control *Rhizoctonia solani* infections in rice [4]. Industrial production of **1** utilizes developed strains of *Streptomyces hygroscopicus* var. *limoneus* or *S. hygroscopicus* var. *jinggangensis* TL01. Structurally, compound **1** comprises an unsaturated aminocyclitol unit, valienamine, a saturated aminocyclitol unit, validamine, and a glucose molecule. Owing to its potential applications and unique chemical structure, extensive efforts have focused on elucidating the biosynthetic pathways of **1**. The biosynthesis of **1** begins with the conversion of sedoheptulose-7-phosphate to 2-*epi*-5-*epi*-valiolone, followed by a series of enzymatic modifications that produce various cyclic intermediates (Fig. 1) [[5], [6], [7], [8], [9]].

In later stages, the pseudoglycosyltransferase ValL (also known as VldE) catalyzes the formation of a *C–N* bond between GDP-valienol (**2**) and validamine-7-phosphate (**3**), resulting in the intermediate validoxylamine A-7′-phosphate (**4**) [10]. This intermediate is subsequently dephosphorylated by the phosphatase ValO to produce validoxylamine A (**5**) [10], which is then glycosylated by the glycosyltransferase ValG to form **1** (Fig. 1) [5].

The pseudoglycosyltransferase ValL/VldE plays a crucial role in **1** biosynthesis. Its substrates, **2** and **3**, are pseudosugar analogues of monosaccharides, in which the ring oxygen is replaced by a methylene group. The unique properties of ValL/VldE have attracted considerable research interest. X-ray crystallography has shown that ValL/VldE adopt the classic "GT-B" fold characteristic of glycosyltransferases [11,12]. Additionally, kinetic isotope effect experiments suggest that ValL/VldE catalyze *C–N* bond formation via a SN_i_-like mechanism [13], analogous to that of OtsA, another GT20 family member that shares structural and sequence similarities with ValL [14,15]. However, unlike the glycosyltransferase reaction catalyzed by OtsA, the ValL-catalyzed reaction does not involve an oxocarbenium ion transition state due to the absence of a ring oxygen in the pseudosugar substrate. Instead, the olefinic group of GDP-valienol may facilitate *C–N* bond formation in a similar manner [13].

Although pseudoglycosyltransferases are believed to have evolved from glycosyltransferases [16], all characterized pseudoglycosyltransferases have been found to catalyze *C–N* bond formation during the biosynthesis of C_7_N aminocyclitols [10,17], whereas most glycosyltransferases catalyze *C–O* bond formation [18]. For example, OtsA catalyzes a condensation reaction between UDP-glucose and glucose-6-phosphate, forming a *C–O* bond, but it cannot catalyze *C–N* bond formation [10]. This specificity may stem from topological differences in key amino acid residues within their *N*-terminal domains [13,19].

Generally, glycosyltransferases exhibit high substrate specificity [20], however, a few of these enzymes have been found to catalyze the formation of multiple types of glycosidic bonds. For example, UGT72B1 catalyzes both *O*- and *N*-glycosidic bond formations [21], whereas UGT74B1 catalyzes both *S*- and *O*-glycosidic bond formations [22]. In addition, UGT74AN3 exhibits remarkable substrate promiscuity, accepting 78 different acceptors and six sugar donors [23]. Given that the glycosylation of natural products usually enhances properties such as solubility, stability, and bioactivity [[24], [25], [26]], glycosyltransferases capable of forming various glycosidic bonds have become a topic of research interest. These glycosyltransferases hold potential in producing glycosylated natural products, thus advancing drug discovery.

In this study, we elucidated the structures of two oxygen-bridged analogues of **1**, namely, validenomycin (**6**) and validomycin (**7**), as well as an oxygen-bridged analogue of **5**, namely, 1,1′-bis-valienol (**8**). Gene inactivation and complementation experiments confirmed that *valL* is involved in the biosynthesis of these oxygen-bridged compounds. Biochemical analysis further revealed that ValL catalyzes a nonglycosidic *C–O* coupling reaction using GDP-valienol (**2**) and valienol-7-phosphate (**9**) as substrates. These findings represent the first discovery of a pseudoglycosyltransferase capable of catalyzing both *C–N* and *C–O* bond formations.

## 2 Materials and methods

### 2.1 Strains, plasmids and media

The detailed information about strains, plasmids, primers, and culture media was listed in Supplementary Tables 1–4. *S*. *hygroscopicus* var. *jinggangensis* TL01 and its derivative strains were used for the fermentation of validamycin A (**1**) and its analogues. *Escherichia coli* strains DH10B, ET12567(pUZ8002) and BL21(DE3) were used as hosts for gene cloning, *E. coli-S. hygroscopicus* bi-parental conjugation and heterologous protein expression, respectively. *E. coli-Streptomyces* shuttle vector pJTU1278 and its derivative plasmids were used for the in-frame gene deletion. Integrative plasmid pPM927 and its derivative plasmids were used for the mutant complementation or gene overexpression. Plasmid pET30a and its derivative plasmids were used for heterologous expression of the target proteins.

### 2.2 General cultural conditions

*S*. *hygroscopicus* var. *jinggangensis* TL01 and its derivative strains were grown on SFM plates at 30 °C for 7 days for sporulation or in TSBY medium at 30 °C for 2 days as seed culture. 5 mL seed culture was inoculated into 50 mL liquid fermentation medium in 250 mL flask and fermented at 37 °C for 4 days. *Rhizoctonia solani* was grown on PDA plates at 30 °C for 3 days, and discs of PDA agar with mycelia were placed on WA plates at 30 °C for 2 days for fungal growth inhibitory assay. *E. coli* strains were cultured in LB medium at 37 °C.

### 2.3 Gene deletion, complementation and overexpression in *S. hygroscopicus* var. *jinggangensis* TL01 and its derivative strains

To construct gene-deleted mutants, the homologous flanking sequences of genes *valL* and *valG* were amplified from genomic DNA of *S. hygroscopicus* var. *jinggangensis* TL01 using the corresponding primers (Supplementary Table 3). The amplicons were digested with *Eco*RI/*Hin*dIII or *Hin*dIII/*Kpn*I and ligated with the *Streptomyces*–*E. coli* shuttle vector pJTU1278 digested with *Eco*RI/*Kpn*I to give pLQ1814 and pLQ1815, and the plasmids were further verified by sequencing. The recombinant plasmids were transferred to *E. coli* ET12567(pUZ8002) and then introduced into strain TL01 by intergeneric conjugation. The exconjugants were streaked to SFM plates with 50 mg/L nalidixic acid and 25 mg/L thiostrepton and incubated for 2–3 days. The exconjugants with thiostrepton resistance were selected and cultured on SFM plates without antibiotic selection, followed by a 7-day incubation. The spores were collected, and colonies that were sensitive to thiostrepton were selected. The double cross-over mutants were verified by PCR amplification.

For the gene complementation and overexpression of the mutant, promoter *kasOp∗* was amplified from plasmid pDR-4-K∗, and genes *valL*, *valG*, *valM*, and *valN* were amplified from the genomic DNA of *S. hygroscopicus* var. *jinggangensis* TL01. The promoter and genes were ligated by overlapping PCR. The PCR-amplified fragment and the linearized pPM927 digested with *Eco*RI were assembled using the NovoRec® plus One step PCR Cloning Kit (NovoProtein). The resulting constructs were transformed into *E. coli* DH10B by CaCl_2_ transformation. After verification by sequencing, the new plasmids, pLQ1820, pLQ1821, pLQ1822 and pLQ1823, were individually introduced into the corresponding derivative strains of TL01 through conjugation, and exconjugants were streaked to SFM plates with 50 mg/L nalidixic acid and 25 mg/L thiostrepton for 2–3 days. Exconjugants with thiostrepton resistance were selected, and the integrated mutants were verified by PCR amplification.

### 2.4 Intergeneric conjugation between *E. coli* and *S. hygroscopicus* var. *jinggangensis* TL01

A culture of *E. coli* ET12567(pUZ8002) containing the recombinant plasmid was grown overnight in LB medium with 25 mg/L chloramphenicol, 50 mg/L kanamycin, and either 100 mg/L ampicillin (for pJTU1278-derived plasmids) or 100 mg/L spectinomycin (for pPM927-derived plasmids). The culture was then inoculated into fresh LB broth at a 1:10 (v/v) ratio and grown for an additional 3 h. Cells were washed three times with 1 mL of LB (about 10^8^–10^9^ CFU/mL). Strain TL01 was cultivated on SFM plate for 7 days. The spores were harvested with 1 mL of 0.05 M TES buffer (pH = 8.0), heated at 50 °C for 10 min, then mixed with 1 mL of 2× YT medium and grown for an additional 3 h. The spores were subsequently washed twice with 1 mL of LB (about 10^8^ CFU/mL). 0.2 mL of spore suspension and 0.5 mL of *E. coli* ET12567(pUZ8002) suspension were mixed and spread on SFM agar plate containing 10 mM MgCl_2_. Then, plates were incubated for 17 h at 30 °C and overlaid with 1 mL LB containing 1 mg nalidixic acid and 0.5 mg thiostrepton. These plates were incubated at 30 °C for another 5–7 days.

### 2.5 Purification and identification of oxygen-bridged analogues of validamycin A and validoxylamine A

For the preparation of oxygen-bridged compounds, 2-L fermentation broth of strain TL01 was harvested by centrifugation. The supernatant was adjusted to pH 3.0–4.0 with oxalic acid. After removing the precipitate by centrifugation, the supernatant was adjusted with NaOH to pH 7.0 and mixed with anion exchange resin D201 (1 g for 10 mL broth) for about 12 h to remove pigments. Then, the mixture was filtered through filter paper, supernatant was then treated with Dowex 50WX8 (H^+^ form) resin and concentrated to 200 mL using vacuum evaporator at 40 °C. The mixture was then subjected to Sephadex LH-20 (2 × 200 cm) column twice. The column was eluted with methanol/water (30:70, v/v), and fractions containing the target compounds were pooled and freeze-dried. The freeze-dried mixture was dissolved in 5 mL of ddH_2_O. Further separation was performed on analytical high-performance liquid chromatography (HPLC, Agilent series 1260, Agilent Technologies, USA) with Agilent ZORBAX SB-Aq (4.6 × 250 mm^2^, particle size 5 μm) at a flow rate of 1 mL/min using an elution buffer of water/methanol (99.5:0.5, v/v). The purified compounds were freeze-dried.

Purified compounds were dissolved in D_2_O, and the one-dimensional (1D) (^1^H, ^13^C, and distortionless enhancement by polarization transfer) and 2D (^1^H–^1^H correlation spectroscopy [COSY], heteronuclear single-quantum correlation spectroscopy [HSQC], hetero-nuclear multiple-bond correlation spectroscopy [HMBC], and nuclear overhauser effect [NOESY]) NMR spectra were collected in D_2_O at 600 MHz (^1^H NMR) and 150 MHz (^13^C NMR) on Bruker Avance III 600 spectrometer (magnetic field strength with 14.09 T). The NMR data processing was performed using the software of MestReNova 9.0.1.

### 2.6 LC-HRMS analysis of the related compounds

The enzymatic reaction mixtures and related compounds were analyzed by liquid chromatography coupled with high-resolution mass spectrometry (LC-HRMS, Agilent 1290–6546 Q-TOF) with Agilent ZORBAX SB-Aq (4.6 × 250 mm, particle size 5 μm) at a flow rate of 0.4 mL/min using an elution buffer composed of (A) Milli-Q water containing 10 mM ammonium formate and (B) methanol with a gradient elution procedure: 0–20 min, 0.5 % B; 20–22 min, 0.5 % B to 95 % B; 22–27 min, 95 % B; 27–28 min, 95 % B to 0.5 % B; 28–38 min, 0.5 % B. The related compounds were detected in negative ion mode. The gas temperature, flow and nebulizer pressure were 325 °C, 8 L/min and 35 psi, respectively. The fragmentor, skimmer and OCT 1 RF Vpp were set at 105, 65 and 750 V, respectively.

### 2.7 Thin-layer chromatography (TLC) analysis of the related compounds

The chromatography plate used was TLC Silica gel 60 F254 Alu (Merck). The mobile phase consisted of *n*-butanol:ethanol:water = 9:7:4 (v/v/v) with the addition of 0.1 % formic acid. Samples were spotted on the plates, 0.5 cm from the lower edge, and allowed to develop in the mobile phase for 45 min. A hair dryer was used for rapid drying of the chromatography plate. Subsequently, a staining solution, comprising 2.5 g of ammonium molybdate tetrahydrate, 1 g of ammonium cerium sulfate, 10 mL of sulfuric acid, and 90 mL of distilled water, was evenly sprayed onto the chromatography plate, followed by heating to 105 °C for visualization.

### 2.8 Preparation of recombinant proteins ValB, ValL, PgmA and ValO

The genes *valB*, *valL*, *pgmA* and *valO* were PCR amplified from strain TL01 using their corresponding primers (Supplementary Table 3). Linearized pET30a was obtained by digestion with *Eco*RI and *Hin*dIII (Thermo Scientific, for *valL*, *pgmA* and *valO*) or *Nde*I and *Hin*dIII (Thermo Scientific, for *valB*). The PCR-amplified fragments and the linearized pET30a were assembled using the NovoRec® plus One step PCR Cloning Kit (NovoProtein). The assembled products were transformed into *E. coli* DH10B by CaCl_2_ transformation. The plasmids were verified by sequencing, and then transferred into *E. coli* BL21(DE3) by CaCl_2_ transformation.

The heterologous expression strains were cultured in 30 mL of LB medium containing 50 mg/L kanamycin. After 12–14 h of cultivation at 37 °C in a shaker (220 rpm), the cultures (10 mL) were transferred to fresh LB medium (1 L) containing 50 mg/L kanamycin and shaken (37 °C, 220 rpm) until OD_600_ reached 0.6–0.8. After cooling to 16 °C, 400 μM isopropyl β-d-1-thiogalactopyranoside (IPTG) was added, and the cultures were continued for 16–20 h at 16 °C with shaking at 180 rpm. The cells were harvested by centrifugation (3200× *g*, 4 °C, 10 min), and the cell pellets were washed with pre-cold Buffer A containing 25 mM Tris-HCl (pH 7.2) and 300 mM NaCl.

Cells were suspended in cold Buffer A and subjected to sonication. The mixture was centrifuged at 13,800 *g* for 30 min at 4 °C, and the supernatant was collected. Ni-NTA resin (GE HealthCare) was employed for immobilizing the target proteins. Nonspecifically bound proteins were removed by washing in turn with 25 mM and 50 mM imidazole dissolved in Buffer A. The target protein was then eluted using 250 mM imidazole in Buffer A and concentrated with Amicon Ultra (MWCO 10–30 K). The imidazole concentration was also reduced to below 12.5 mM using ultrafiltration. The purified protein was validated by SDS-PAGE, and the protein concentration was determined using NanoDrop One (Thermo Scientific).

### 2.9 In vitro enzymatic assays

For the enzymatic assay of ValB and PgmA, a 30 μL reaction mixture, containing 25 mM Tris-HCl (pH 7.5), 10 mM GTP, 2.5 mM valienol-7-phosphate (**9**) and 2 μM of each enzyme, was incubated at 30 °C for 6 h. The reaction mixtures with boiled ValB or PgmA were set as controls. The reaction was stopped by adding two volumes of methanol, followed by vigorous vortex to denature proteins. The mixture was centrifuged at 13,800 *g* for 20 min, and the supernatant was filtered through a 0.22 μm filter and subsequently subjected to LC-HRMS analysis.

For the enzymatic assay of ValB, PgmA and ValL, a 30 μL reaction mixture, containing 25 mM Tris-HCl (pH 7.5), 10 mM MgCl_2_, 10 mM GTP, 2.5 mM **9**, and 2 μM of each enzyme, was incubated at 30 °C for 6 h. The reaction mixture with boiled ValL was set as control. The reaction was stopped by adding two volumes of methanol, followed by vigorous vortex to denature proteins. The mixture was centrifuged at 13,800 *g* for 20 min, and the supernatant was filtered through a 0.22 μm filter and subsequently subjected to LC-HRMS analysis.

For the enzymatic assay of ValO, a 30 μL reaction of ValB, PgmA and ValL containing 25 mM Tris-HCl (pH 7.5), 10 mM MgCl_2_, 10 mM GTP, 2.5 mM **9**, and 2 μM of each enzyme, was incubated at 30 °C for 6 h, the reaction was stopped by adding two volumes of methanol, followed by vigorous vortex to denature proteins. The freeze-dried reaction residue was then dissolved in 30-μL dd water with 2 μM ValO and incubated at 30 °C for 2 h. The reaction was stopped by heating at 75 °C for 5 min, followed by vigorous vortex to denature proteins. The mixture was centrifuged at 13,800 *g* for 20 min, and the supernatant was filtered through a 0.22 μm filter and subsequently subjected to LC-HRMS analysis.

### 2.10 Fungal growth inhibitory assay of validamycin A and its oxygen-bridged analogues

WA medium was plated into Petri dishes. Filter papers containing compounds were placed in the center of the plates. Discs of PDA agar with mycelia of *Rhizoctonia solani* were placed at the corners of the plates as indicator strain for bioassay of the compounds. The plates were incubated at 30 °C for 2 days. Antifungal activity of compounds was determined based on their ability to inhibit the expansion of the fungal mycelia on the Petri dishes, water and validamycin A (**1**) were set as negative and positive control, respectively.

### 2.11 Determination of *Ki* values for validoxylamine A and 1,1′-bis-valienol

The inhibition constant (*Ki*) values for validoxylamine A (**5**) and 1,1′-bis-valienol (**8**) against pig-kidney trehalase were determined using a stopped assay, the initial reaction rate (*V*_0_) determined by quantifying of glucose production using glucose oxidase/peroxidase kit (Leagene, Beijing, China). Assays were performed at 37 °C in PBS buffer (pH 7.0). Trehalose concentrations included 0.15, 0.3, 0.6, 1.2, 2.6, 6.2 and 10.5 mM. Compound **5** concentrations were 0, 1.87, 3.73, and 7.49 nM, and **8** concentrations were 0, 18.7, 37.4, and 74.9 μM as inhibitors. Trehalase was present at a final concentration of 1 μM. 50 μL aliquots of the reaction mixture were taken at intervals over 15 min. Each aliquot was boiled for 5 min, followed by the addition of 150 μL of the glucose oxidase/peroxidase solution. Trehalase was pre-incubated with each of the inhibitors for 10 min prior to the start of the assay to prevent any complications with slow-onset inhibition.

The substrate concentrations [S], inhibitor concentrations [I], and their corresponding initial reaction rates [*V*_0_] for different groups were recorded, and inhibition constants *Ki* were fitted using Graphpad Prism 8.3.0.

### 2.12 Construction of the phylogenetic tree

The homologs of ValL were identified using a BlastP search with standard parameters. The maximum number of target sequences was changed to 250. The phylogenetic tree was constructed by Geneious Prime (version 2023.2.1) using Jukes-Cantor genetic distance model and Neighbor-Joining tree building method. The constructed tree was edited by iTOL [27].

## 3 Results

### 3.1 Discovery and structure elucidation of the oxygen-bridged analogues of validamycin A

**Fig. 2:**
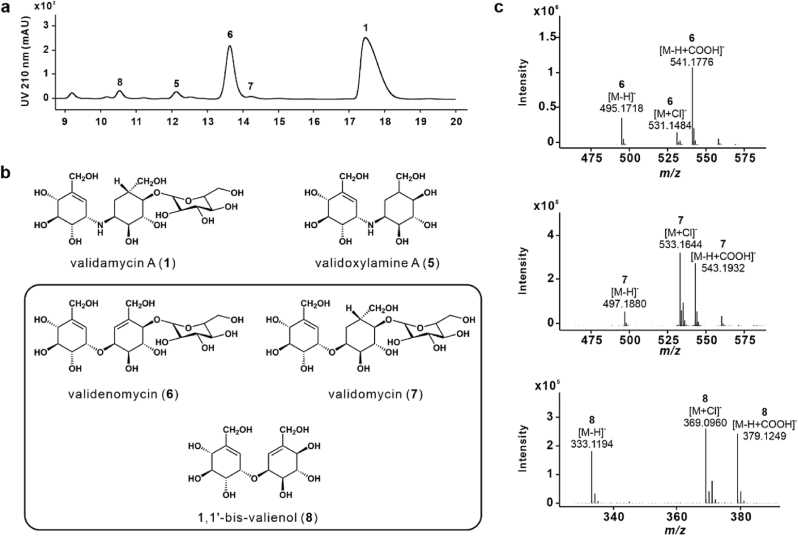
**Discovery and structure elucidation of three novel oxygen-bridged analogues of validamycin A (1). a**, HPLC profiles of the fermentation broth from strain TL01, monitored at 210 nm. **b**, Chemical structures of validamycin A (**1**), validoxylamine A (**5**), validenomycin (**6**), validomycin (**7**) and 1,1′-bis-valienol (**8**). The newly identified oxygen-bridged compounds in this study are highlighted with black boxes. **c**, HRMS analysis of compounds **6**, **7** and **8**.

During a detailed analysis of the chemical components of validamycins in the fermentation broth of the industrial strain *S. hygroscopicus* var. *jinggangensis* TL01 using liquid chromatography coupled with high-resolution mass spectrometry (LC-HRMS), two unexpected compounds with *m/z* values of 495.1718 [M − H]^−^ and 497.1880 [M − H]^−^ (Fig. 2a and c) were observed besides validamycin A (**1**) and validoxylamine A (**5**). Given that the molecular weights of these two compounds differ by only ±1 from that of **1**, they are likely intriguing analogues of **1**, designated as **6** and **7**, respectively (Fig. 2b). Moreover, the corresponding deglycosylated products of **6**—designated as **8** (Fig. 2b)—were detected using HRMS with a *m/z* value of 333.1194 [M − H]^−^ (Fig. 2c). Compound **8** was structurally similar to **5**.

To determine the chemical structures of the newly discovered compounds, 2-L cultures of strain TL01 were fermented. The fermentation broth was collected via centrifugation, and the supernatant was then treated with Dowex 50WX8 (H^+^ form) resin and continuously subjected to Sephadex LH-20 column chromatography and HPLC to isolate the target compounds. Using thin-layer chromatography and MS, we obtained three new compounds, designated as **6**, **7**, and **8**. Their chemical structures were subsequently elucidated using 1D and 2D NMR spectroscopy and HRMS data analysis. (Supplementary Tables 5–12, Supplementary Figs. 1–4).

The ^1^H NMR spectrum of **6** (Supplementary Tables 7 and 8, Supplementary Fig. 2) showed two olefinic proton signals [6.13 (d, *J* = 5.1 Hz) and 6.04 (d, *J* = 5.1 Hz)] and an anomeric proton signal [4.66 (d, *J* = 8.0 Hz)], along with other proton signals as assigned to the core cyclitol and glucose units. Notably, two cyclitol proton signals [4.36 (t, *J* = 4.7 Hz) and 4.35 (t, *J* = 4.7 Hz)] suggested that the cyclitol moieties were connected via an oxygen bridge. The ^13^C NMR spectrum displayed 20 carbon signals, including four olefinic carbon signals [142.45 (C5), 140.16, (C5′), 123.23, (C6′), and 120.96 (C6)] and an anomeric carbon signal [103.21, (C1″)]. This pattern supported the presence of two valienol moieties and a glucose unit, aligning with the mass data (*m/z* value of 495.1718 [M − H]^−^). Further, 2D NMR analysis indicated the attachment of glucose at the C-4′ position. Based on the NMR spectra and molecular mass of **6**, its structure was confirmed and was named validenomycin (**6**).

The NMR spectra of **7** resembled those of **6** (Supplementary Tables 9 and 10, Supplementary Fig. 3). A comparison between the ^1^H NMR spectra of compounds **7** and **6** revealed that **7** had two methylene proton signals [1.27 (t, *J* = 13.3 Hz) and 2.16 (m)] and only one olefinic proton signal [6.06 (d, *J* = 5.1 Hz)]. Additionally, the ^13^C NMR spectrum of **7** showed a secondary carbon signal [26.87 (C6′)], a tertiary carbon signal [37.54 (C5′)], and two olefinic carbon signals [142.46 (C5), 120.96 (C6)]. These features suggested a structural similarity between **7** and **6**, with differences primarily observed in the respective degrees of unsaturation. Specifically, **7** comprised valienol, validol, and glucose moieties, consistent with its mass data (*m/z* value of 497.1880 [M − H]^−^). Based on the molecular mass, the proposed structure of compound **7** was confirmed, and it was named validomycin (**7**).

Compound **8** exhibited relatively simple ^1^H and ^13^C NMR spectra (Supplementary Tables 11 and 12, Supplementary Fig. 4), with only seven carbon signals observed in the ^13^C NMR spectrum. The presence of an olefinic proton signal at 6.06 ppm (brd, *J* = 4.8 Hz) in the ^1^H NMR spectrum, along with two olefinic carbon signals [142.40 (C5) and 121.01 (C6)] in the ^13^C NMR spectrum, indicated the presence of an unsaturated cyclitol moiety, valienol. Given the molecular mass (*m/z* value of 333.1194 [M − H]^−^), compound **8** was proposed to be a symmetrical dimer of valienol, namely 1,1′-bis-valienol (**8**). This inference was further supported by a cyclic proton signal at 4.36 ppm (t, *J* = 4.7 Hz), confirming the presence of an oxygen bridge connecting the two valienol moieties.

### 3.2 ValL is involved in the production of validamycin A and its oxygen-bridged analogues

**Fig. 3:**
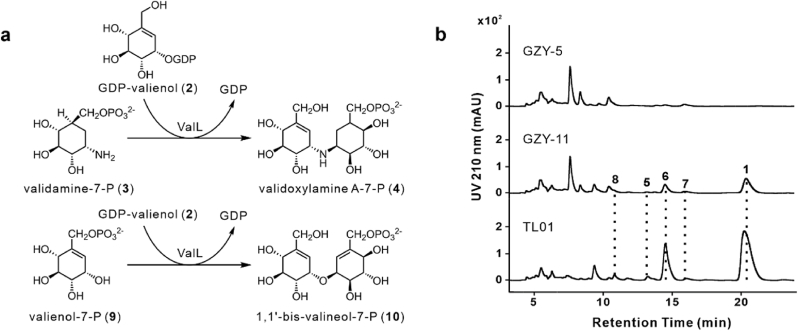
**ValL is involved in the biosynthesis of validamycin A (1) and its oxygen-bridged analogues. a**, ValL catalyzes the formation of validoxylamine A-7′-phosphate (**4**) via a *C–N* bond between GDP-valienol (**2**) and validamine-7-phosphate (**3**), analogous to the *C–O* bond formation in 1,1′-bis-valienol-7-phosphate (**10**) between GDP-valienol (**2**) and valienol-7-phosphate (**9**). **b**, HPLC profiles of the fermentation broth from strain TL01, GZY-5, and GZY-11, monitored at 210 nm. GZY-5: TL01*ΔvalL*. GZY-11: TL01*ΔvalL*:pLQ1820.

ValL functions as an essential enzyme in the biosynthesis of validamycin A (**1**) and validoxylamine A (**5**), catalyzing the *C–N* bond coupling reaction between GDP-valienol (**2**) and validamine-7-phosphate (**3**) to produce validoxylamine A-7′-phosphate (**4**) [10]. Given that the only structural difference between 1,1′-bis-valienol (**8**) and **5** lies in the oxygen bridge and degree of unsaturation, ValL was considered to participate in the biosynthesis of oxygen-bridged analogues of **1** and **5** (Fig. 3a).

To investigate the involvement of ValL in this *C–O* bond coupling reaction, a 1.28 kb DNA segment of *valL* was deleted from strain TL01 through double-crossover recombination. This deletion was carried out using the pJTU1278-derived plasmid pLQ1814 [28], which included polymerase chain reaction (PCR)-amplified 1.47 kb upstream and 1.67 kb downstream flanking sequences of *valL*. Plasmid pLQ1814 was introduced into strain TL01 via conjugation, and the resulting double-crossover recombinant mutant was named GZY-5 (Supplementary Fig. 5). Mutant strain GZY-5 was cultured at 37 °C in fermentation medium, and HPLC analysis of the fermentation broth showed that the oxygen-bridged compounds **6**, **7** and **8** disappeared. As expected, **1** and **5** also disappeared from the fermentation broth (Fig. 3b).

To further validate the role of *valL* in the biosynthesis of these oxygen-bridged analogues, gene complementation was performed. The *valL* gene was amplified and cloned into the integrative vector pPM927 under the control of the *kasO*p∗ promoter to generate pLQ1820 [29,30]. The plasmid was then introduced into the *valL*-deleted mutant GZY-5 through conjugation, generating the recombinant strain GZY-11 (Supplementary Fig. 6). The GZY-11 strain was cultured at 37 °C in fermentation medium, and HRMS analysis of the fermentation broth revealed a partial restoration of the oxygen-bridged compounds **6**, **7**, and **8** (Fig. 3b). Thus, the combination of gene deletion and complementation experiments provided convincing evidence to indicate the involvement of *valL* in the biosynthesis of both **1**, **5** and their oxygen-bridged analogues **6**, **7**, and **8**.

### 3.3 ValL catalyzes C–O bond formation between valienol-7-phosphate and GDP-valienol

**Fig. 4:**
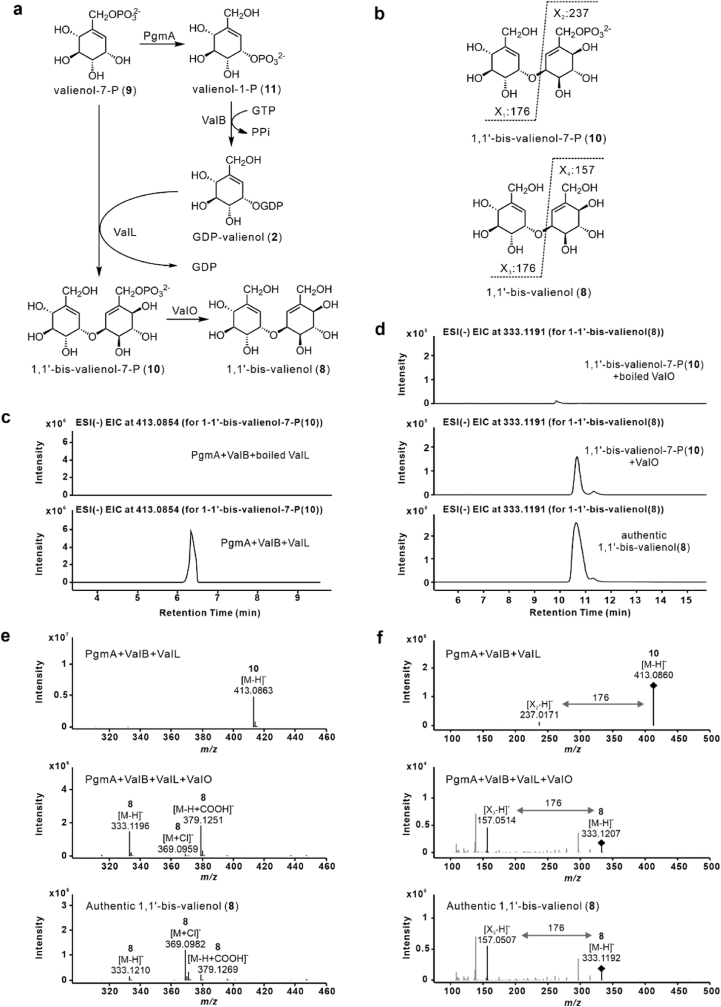
**HRMS and HRMS/MS analysis of ValL and ValO catalyzed reaction products. a**, Reaction scheme of PgmA, ValB, ValL and ValO. **b**, Chemical structures of 1,1′-bis-valienol (**8**) and 1,1′-bis-valienol-7-phosphate (**10**). **c**, Partial LC-HRMS extracted ion chromatograms (EIC, negative ion mode) of the reaction catalyzed by PgmA, ValB, and ValL using valienol-7-phosphate (**9**) and GTP as substrates, with a boiled ValL reaction serving as a negative control. Both chromatograms are the extraction of the corresponding calculated exact mass for **10** (*m/z* 413.0854 [M − H]^–^). **d**, Partial LC-HRMS EIC chromatograms (negative ion mode) of the ValO reaction with **10**, alongside a boiled ValO negative control and a compound **8** standard. All chromatograms are the extraction of the corresponding calculated exact mass for **8** (*m/z* 333.1191 [M − H]^–^). **e**, HRMS analysis of reaction products catalyzed by PgmA, ValB and ValL; PgmA, ValB, ValL and ValO; and compound **8** standard. **f**, HRMS/MS analysis of reaction products catalyzed by PgmA, ValB and ValL; PgmA, ValB, ValL and ValO; and compound **8** standard.

To validate the function of ValL in catalyzing condensation reactions to form pseudoglycosyl *C–O* bonds, biochemical assays were performed using GDP-valienol (**2**) and valienol-7-phosphate (**9**) as the donor and acceptor substrates, respectively. Owing to the instability of compound **2**, it was produced via a coupling reaction with ValB, PgmA, **9**, and GTP (Fig. 4a). Phosphoglucomutase PgmA typically catalyzes the conversion between glucose-6-phosphate and glucose-1-phosphate [31]. Moreover, a phosphoglucomutase from *Sphingomonas paucimobilis* ATCC 31461 also exhibits catalytic activity with substrate promiscuity [32]. Given the structural similarity between **9** and glucose-6-phosphate, in this reaction, PgmA is proposed to convert **9** into valienol-1-phosphate (**11**), and this intermediate could be subsequently converted to GDP-valienol (**2**) by the nucleotidyltransferase ValB [33].

The genes *valB*, *pgmA*, and *valL* were amplified via PCR and cloned into the expression vector pET-30a, the resulting plasmids were then introduced into *Escherichia coli* BL21(DE3). After 16 h of culture at 16 °C, we confirmed the successful expression of recombinant ValB, PgmA, and ValL. The harvested cells were lysed via ultrasonication and centrifuged, with the resulting supernatant being purified using nickel-nitrilotriacetic acid (Ni-NTA) affinity chromatography (Supplementary Fig. 7). A 30 μL reaction mixture, containing 25 mM Tris-HCl (pH 7.5), 10 mM MgCl_2_, 10 mM GTP, 2.5 mM **9**, and 2 μM each of ValB and PgmA, was incubated at 30 °C for 6 h. A reaction mixture containing boiled ValB or PgmA was used as a negative control. After reaction quenching with 60 μL methanol and protein removal, the products were analyzed using LC-HRMS.

HRMS analysis of the product obtained from the ValB and PgmA reaction with substrates **9** and GTP confirmed the formation of **2**, showing an [M − H]^−^ ion at *m/z* 600.0748, consistent with the calculated *m/z* of 600.075 [M − H]^−^. high-resolution tandem mass spectrometry (HRMS/MS) analysis yielded fragment ions at *m/z* values of 442.0178 [M − H]^−^ and 362.0511 [M − H]^−^, in alignment with reported data, thereby confirming the reaction products (Supplementary Fig. 8). Subsequently, recombinant ValL was added into ValB and PgmA coupling reactions. A 30 μL reaction mixture, containing 25 mM Tris-HCl (pH 7.5), 10 mM MgCl_2_, 10 mM GTP, 2.5 mM **9**, and 2 μM each of ValB, PgmA and ValL, was incubated at 30 °C for 6 h. As a negative control, boiled ValL was also prepared and included in a separate reaction. The reactions were quenched using methanol, and proteins were removed via centrifugation, then the products were analyzed using LC-HRMS. HRMS analysis of the reaction products revealed the formation of a new product with an *m*/*z* of 413.0862 [M − H]^−^ (Fig. 4c and e), consistent with the calculated *m*/*z* of 413.0854 [M − H]^−^ for the predicted product **10**.

Further HRMS/MS analysis of the ValL product and the authentic **8** standard revealed distinct fragment ions. The ValL product generated a fragment ion at *m/z* 237.0171 [M − H]^−^, whereas the authentic **8** produced a fragment ion at *m/z* 157.0507 [M − H]^−^. This 80 amu difference corresponds to the molecular weight of a phosphate group, indicating that compared with compound **8**, the ValL product contains an additional phosphate group (Fig. 4f).

### 3.4 ValO catalyzes the dephosphorylation of 1,1′-bis-valienol-7-phosphate

ValO, a phosphatase, catalyzes the dephosphorylation of validoxylamine A-7′-phosphate (**4**) to generate validoxylamine A (**5**) during the biosynthesis of validamycin A (**1**) [10]. Given the structural similarity between 1,1′-bis-valienol-7-phosphate (**10**) and **4**, we reasonably hypothesized that ValO might also catalyze the dephosphorylation of **10** to generate 1,1′-bis-valienol (**8**).

To test this hypothesis, the *valO* gene was amplified via PCR and cloned into the expression vector pET-30a, which was then introduced into *E. coli* BL21(DE3). After a 16 h culture at 16 °C, recombinant ValO was successfully expressed. Cells were harvested, lysed by ultrasonication, and centrifuged. The supernatant was then purified using Ni-NTA affinity chromatography (Supplementary Fig. 7).

For the biochemical assay of ValO, a 30-μL reaction mixture containing 25 mM Tris-HCl (pH 7.5), 10 mM MgCl_2_, 2.5 mM **9**, 10 mM GTP and 2 μM each of ValB, PgmA and ValL, was incubated at 30 °C for 6 h. The reaction was quenched using methanol, after removing the proteins, the supernatant was freeze-dried and then dissolved in 30-μL double-distilled water containing 2 μM ValO. This mixture was incubated at 30 °C for 2 h, then heated at 75 °C for 5 min to terminate the reaction. The reaction products were analyzed by LC-HRMS in negative mode, revealing the dephosphorylated product **8** (*m/z* = 333.1196 [M − H]^−^) (Fig. 4d and e). HRMS/MS analysis of the product revealed a fragment ion at *m/z* 157.0514 [M − H]^−^, which was identical to the fragment ion of authentic **8** at 157.0507 [M − H]^−^ (Fig. 4f), thereby confirming that ValO catalyzes the dephosphorylation of **10** to yield the final product, **8**.

### 3.5 ValG catalyzes the glycosylation of 1,1′-bis-valienol

**Fig. 5:**
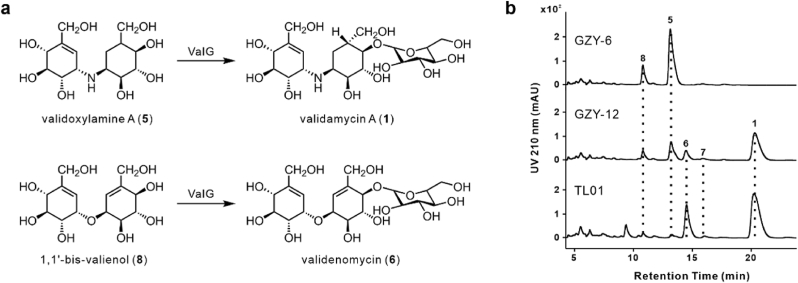
**ValG is responsible for the glycosylation of 1,1′-bis-valienol (8) to produce validenomycin (6). a**, ValG catalyzes the glycosylation of validoxylamine A (**5**) to generate validamycin A (**1**), similar to the glycosylation of 1,1′-bis-valienol (**8**) to form validenomycin (**6**). **b**, HPLC profiles of the fermentation broth from strain TL01, GZY-6, and GZY-12, monitored at 210 nm. GZY-6: TL01*ΔvalG*. GZY-12: TL01*ΔvalG*:pLQ1821.

ValG is a glucosyltransferase that catalyzes the glycosylation of validoxylamine A (**5**) at the C4′ position to produce validamycin A (**1**) [34]. Since validenomycin (**6**) and validomycin (**7**) also feature a glucose moiety attached at the C4′ position, it is likely that glucosyltransferase ValG is similarly involved in the biosynthesis of **6** and **7** (Fig. 5a).

To ascertain the function of ValG in the biosynthesis of compounds **6** and **7**, we deleted a 1.09 kb segment of *valG* in strain TL01 using the pJTU1278-derived plasmid pLQ1815 [28], which contained PCR-amplified flanking sequences of *valG*. Plasmid pLQ1815 was introduced into strain TL01 via conjugation, and the resulting double-crossover mutant, GZY-6 (Supplementary Fig. 9). HPLC analysis of the fermentation broth from GZY-6 showed that the biosynthesis of **6** and **7** was disrupted (Fig. 5b).

Gene complementation was subsequently performed by reintroducing *ValG*, under the control of the *kasO*p∗ promoter, into strain GZY-6, thereby generating the recombinant strain GZY-12 [29,30] (Supplementary Fig. 10). The mutant strain GZY-12 was cultured at 37 °C, and HPLC analysis of the fermentation broth of GZY-12 revealed the restoration of **6** and **7** production (Fig. 5b). These gene deletion and complementation experiments clearly demonstrate that glucosyltransferase ValG plays a key role in the biosynthesis of compounds **6** and **7**.

### 3.6 Bioactivity assessment of the oxygen-bridged compounds

**Fig. 6:**
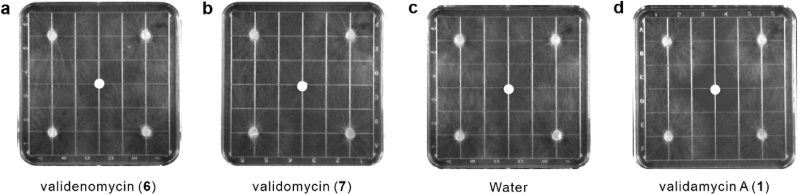
**Fungal growth inhibitory assays of validenomycin (6) and validomycin (7). a**, Fungal growth inhibitory assay of validenomycin (**6**). **b**, Fungal growth inhibitory assay of validomycin (**7**). **c**, Fungal growth inhibitory assay of water. **d**, Fungal growth inhibitory assay of validamycin A (**1**). In each assay, the tested compounds were applied to the filter paper placed at the center of the plates, while fungal cultures were inoculated as agar plugs positioned at the plate corners.

Given the structural similarities among validenomycin (**6**), validomycin (**7**), and validamycin A (**1**), which exhibit potent antifungal activity [35], it was hypothesized that compound **6** and **7** might possess similar bioactivities. Antifungal activity assays were conducted for **6** and **7** against *R. solani* using an agar plug assay. In this setup, the fungus was inoculated as an agar plug placed at the corners of the plates, while filter papers containing the test compounds were positioned in the center, with **1** serving as a positive control and water serving as a negative control. The antifungal activities of the compounds were determined based on their ability to inhibit the expansion of fungal mycelia in Petri dishes. The results revealed that neither **6** nor **7** exhibited significant antifungal activity when compared with that of **1** (Fig. 6).

**1** is hydrolyzed by β-glucosidase during its transport into fungal mycelia, resulting in the formation of validoxylamine A (**5**), a potent trehalase inhibitor [36]. Trehalase is responsible for catalyzing the hydrolysis of trehalose, generating glucose for energy production or other metabolic uses [37,38]. Accordingly, the inhibition of trehalase by **5** may disrupt fungal metabolism, potentially leading to death [39].

To investigate whether 1,1′-bis-valienol (**8**), the deglycosylated product of **6**, exhibits inhibitory activity against trehalase, the inhibitory constants (*Ki*) of **8** were determined through an assay, in which glucose production was measured using a glucose oxidase/peroxidase kit (Leagene, Beijing, China) following trehalose hydrolysis, and **5** served as the positive control. We obtained *Ki* values of 39.3 μM and 2.6 nM for **8** and **5**, respectively. These results indicated that **8** demonstrated only 0.0066 % of the inhibitory activity toward trehalase compared with **5** (Supplementary Fig. 11). These findings elucidated the limited antifungal activities of **6** and **7**.

## 4 Discussion

**Fig. 7:**
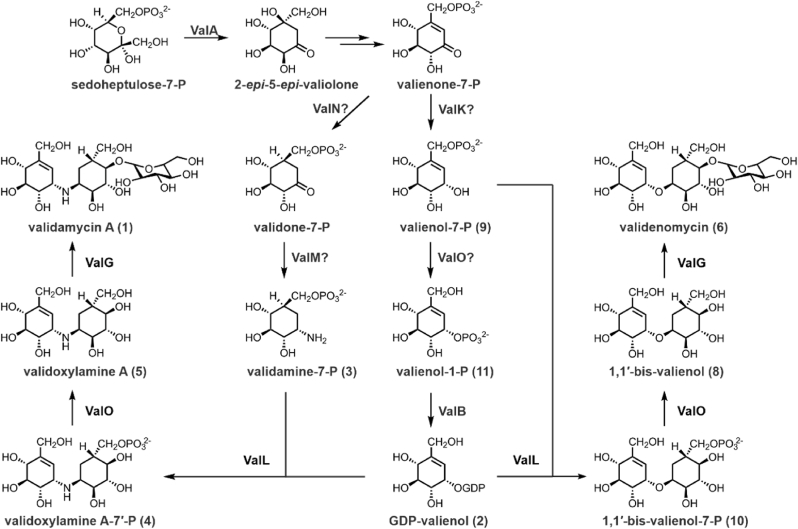
**Biosynthetic pathway of the oxygen-bridged compound validenomycin (6) closely resembles that of validamycin A (1).** The biosynthesis of validenomycin (**6**) originates from sedoheptulose-7-phosphate and follows the same early steps as validamycin A (**1**). The overall biosynthetic pathway of validenomycin (**6**) and validamycin A (**1**) differs only in the branching step mediated by ValL. Enzymes highlighted with black ovals are those discussed in detail in the Results section. Question marks indicate steps in the pathway where the responsible enzyme has not been definitively identified.

Through gene knockout, complementation studies, and in vitro enzyme assays, we established that the validoxylamine A-7′-phosphate (**4**) synthase ValL catalyzes a condensation reaction between GDP-valienol (**2**) and valienol-7-phosphate (**9**), thereby generating 1,1′-bis-valienol-7-phosphate (**10**). This intermediate is subsequently dephosphorylated by ValO and glycosylated by ValG to produce the final product, validenomycin (**6**), mirroring the biosynthetic pathway of validamycin A (**1**) (Fig. 7) [10,11]. Notably, the observed substrate flexibility of ValG indicates that such enzymatic activity could be leveraged to broaden the range of glycosylated products, potentially facilitating the creation of novel bioactive compounds.

Similar to the bifunctional *N*- and *O-*glycosyltransferase UGT72B1 [21], ValL functions as a bifunctional *N*- and *O*-pseudoglycosyltransferase during the biosynthesis of compounds **1**, **6**, and **7**. This enzymatic promiscuity highlights the broader substrate recognition and catalytic versatility of ValL. Interestingly, a higher accumulation of **6** than that of **7** was observed in the fermentation products, despite **7** being structurally more similar to the primary product **1**. This disparity may be attributed to the low levels of the intermediate validol-7-phosphate during fermentation, coupled with the greater catalytic efficiency of ValL for the condensation of GDP-valienol (**2**) with valienol-7-phosphate (**9**) compared with that of validol-7-phosphate.

Antifungal assays against *R. solani* revealed that both validenomycin (**6**) and validomycin (**7**) were less effective against this fungus than that of validamycin A (**1**). Enzyme inhibition assays indicated that 1,1′-bis-valienol (**8**), the deglycosylated product of **6**, exhibited only 0.0066 % inhibitory activity toward trehalase compared with that of validoxylamine A (**5**). These findings highlight the critical role of the imino group (-NH) in the antifungal activity of compound **1**, as it likely forms electrostatic interactions with the acidic amino residues of trehalase, enhancing its binding affinity [1]. Additionally, the adjacent nonglycosidic *C–N* bond may prevent enzymatic hydrolysis [40]. These findings thus provide insights into the mechanisms underlying the inhibition of trehalase, and may therefore contribute to the development of more effective antifungal agents.

To explore the evolutionary characteristics of ValL and its homologs, a Basic Local Alignment Search Tool (BLAST) analysis was performed using the ValL sequence against the National Center for Biotechnology Information database. This analysis identified 250 homologous protein sequences. A phylogenetic tree was constructed based on these sequences (Supplementary Fig. 12). The phylogenetic tree revealed two distinct clades, the first of which (shaded purple) includes ValL and other putative pseudoglycosyltransferases, such as SalC, involved in the biosynthesis of aminocyclitol salbostatin [41]. These putative pseudoglycosyltransferase genes are clustered together with sugar phosphate cyclase genes (similar to *valA*), nucleotide transferases (similar to *valB*), and transaminases (similar to *valM*), indicating their involvement in the biosynthesis of pseudo-oligosaccharides such as validamycin A (**1**). In contrast, genes in the second clade (shaded yellow) did not cluster within biosynthesis gene clusters (BGCs); rather, they seemed to represent common trehalose-6-phosphate synthases.

These findings are intriguing, as we initially hypothesized that evolutionary intermediates might exist between ValL and trehalose-6-phosphate synthases (such as OtsA) in certain pseudo-oligosaccharide BGCs that lack transaminases. These BGCs could potentially produce pseudo-oligosaccharides without amino groups. However, in all ValL homologous proteins identified using BLAST for protein analysis, we consistently identified transaminases within their gene clusters. Bioactivity assays provided further evidence that pseudo-oligosaccharides containing *C–N* bonds have a higher bioactivity than those containing *C–O* bonds. This suggested that gene clusters responsible for producing amino-containing pseudo-oligosaccharides may provide a selective advantage to their host organisms. Moreover, the findings of biochemical assays revealed that ValL can catalyze the formation of *C–O* bond pseudo-oligosaccharides, thereby offering potential insights into the evolutionary trajectory of pseudoglycotransferases.

In previous studies, validoxylamine A (**5**) was believed to be the primary by-product of validamycin A (**1**) production in fermentation broth. Glycosylation of **8** using UDP-glucose significantly increased the yield of **1** [42]. However, in this study, we identified oxygen-bridged analogues of **1**, namely validenomycin (**6**) and validomycin (**7**). Using ultraviolet–visible spectroscopy, we determined that compound **6** accounts for approximately 30 % of the total **1** produced. In our study, the accumulation of a large quantity of oxygen-bridged byproducts suggested inefficient utilization of the key intermediate valienol-7-phosphate (**9**) in **1** biosynthesis. Thus, we reasoned that redirecting the excess compound **9** toward **1** production could potentially increase the yield of **1**. To assess this possibility, we attempted to overexpress *valM* and *valN* to enhance the production of validamine-7-phosphate (**3**), aiming to direct ValL toward synthesizing validoxylamine A-7′-phosphate (**4**) instead of 1,1′-bis-valienol-7-phosphate (**10**). However, this strategy did not yield a significant improvement in **1** production or a reduction in its oxygen-bridged analogues (Supplementary Fig. 13). These results may be attributed to the broad substrate specificity of ValL, which limits its utilization of **3** despite its increased availability.

In conclusion, we have identified a novel class of oxygen-bridged validamycin analogues and investigated their structures, physiological activities, and biosynthetic processes. The validoxylamine A-7′-phosphate (**4**) synthase ValL was established to catalyze the formation of these oxygen-bridged pseudosugars, offering new insights into the catalytic mechanisms of pseudoglycosyltransferases. Given that these oxygen-bridged byproducts consume significant amounts of valienol-7-phosphate (**9**), the precursor of **1**, future studies should focus on minimizing the formation of these by-products to enhance **1** production. Overall, this study provides new insights into strategies for improving the yield of **1**, as well as the catalytic mechanisms and evolutionary characteristics of pseudoglycosyltransferases.

## CRediT authorship contribution statement

**Ziyue Guo:** Writing – review & editing, Writing – original draft, Methodology, Investigation, Formal analysis, Data curation, Conceptualization. **Xin Zhang:** Writing – original draft, Formal analysis, Data curation. **Lin Zhou:** Writing – review & editing, Methodology, Data curation. **Qungang Huang:** Writing – review & editing, Methodology, Formal analysis. **Qianjin Kang:** Writing – review & editing, Writing – original draft, Methodology, Data curation. **Linquan Bai:** Writing – review & editing, Supervision, Methodology, Funding acquisition, Data curation, Conceptualization.

## Declaration of competing interest

The author Linquan Bai is an Editorial Board Member for Synthetic and Systems Biotechnology and was not involved in the editorial review or the decision to publish this article. Other authors declare that they have no known competing financial interests or personal relationships that could have appeared to influence the work reported in this paper.
