# Supplementary material for: Nonglycosidic C–O bond formation catalyzed by a bifunctional pseudoglycosyltransferase ValL
Source: Synth Syst Biotechnol. 2025 Apr 17;10(3):846–57. doi: 10.1016/j.synbio.2025.04.007 (PMC12041759; doi:10.1016/j.synbio.2025.04.007)
Supplement: Multimedia component 1 [file mmc1.docx]

**Supplementary information**

**Nonglycosidic *C-O* Bond Formation Catalyzed by a Bifunctional Pseudoglycosyltransferase ValL**

*Ziyue Guo^1^, Xin Zhang^1^, Lin Zhou^1^, Qungang Huang^1^, Qianjin Kang^1,2^*, Linquan Bai^1,2^**

^1^State Key Laboratory of Microbial Metabolism, School of Life Sciences and Biotechnology, Shanghai Jiao Tong University, Shanghai 200240, China.

^2^College of Life Science and Technology, Tarim University, Alar 843300, Xinjiang, China.

*Corresponding authors. E-mails: qjkang@sjtu.edu.cn, bailq@sjtu.edu.cn

**Table of Contents**

[**Supplementary information** 1](#_Toc182834316)

[**SUPPLEMENTARY TABLES** 4](#_Toc182834317)

[**Supplementary Table 1 Strains used in this study and their properties and sources** 4](#_Toc182834318)

[**Supplementary Table 2 Plasmids used in this study and their properties and sources** 5](#_Toc182834319)

[**Supplementary Table 3 Primers used in strain constructions** 6](#_Toc182834320)

[**Supplementary Table 4 Medium components** 8](#_Toc182834321)

[**Supplementary Table 5 HR-MS data of investigated compounds in negative ionization mode** 9](#_Toc182834322)

[**Supplementary Table 6 HR-MS/MS data of investigated compounds** 10](#_Toc182834323)

[**Supplementary Table 7 ^1^H-NMR Data for validenomycin (D_2_O, 600 MHz)** 11](#_Toc182834324)

[**Supplementary Table 8 ^13^C-NMR Data for validenomycin (D_2_O, 150 MHz)** 12](#_Toc182834325)

[**Supplementary Table 9 ^1^H-NMR Data for validomycin (D_2_O, 600 MHz)** 13](#_Toc182834326)

[**Supplementary Table 10 ^13^C-NMR Data for validomycin (D_2_O, 150 MHz)** 14](#_Toc182834327)

[**Supplementary Table 11 ^1^H-NMR Data for 1,1′-bis-valienol (D_2_O, 600 MHz)** 15](#_Toc182834328)

[**Supplementary Table 12 ^13^C-NMR Data for 1,1′-bis-valienol (D_2_O, 150 MHz)** 16](#_Toc182834329)

[**SUPPLEMENTARY FIGURES** 17](#_Toc182834330)

[**Supplementary Figure 1. Chemical structures of validamycin A and its derivatives involved in this study.** 17](#_Toc182834331)

[**Supplementary Figure 2. NMR analysis of validenomycin.** 21](#_Toc182834332)

[**Supplementary Figure 3. NMR analysis of validomycin.** 25](#_Toc182834333)

[**Supplementary Figure 4. NMR analysis of 1,1′-bis-valienol.** 29](#_Toc182834334)

[**Supplementary Figure 5. Construction of GZY-5 strain.** 30](#_Toc182834335)

[**Supplementary Figure 6. Construction of GZY-11 strain.** 31](#_Toc182834336)

[**Supplementary Figure 7. Overexpression of recombinant His-tagged target proteins in *E. coli* BL21(DE3) and analysis by SDS-PAGE.** 32](#_Toc182834337)

[**Supplementary Figure 8. HR-MS and HR-MS/MS analysis of PgmA and ValB reaction products.** 33](#_Toc182834338)

[**Supplementary Figure 9. Construction of GZY-6 strain.** 34](#_Toc182834339)

[**Supplementary Figure 10. Construction of GZY-12 strain.** 35](#_Toc182834340)

[**Supplementary Figure 11. Inhibitory kinetics of validoxylamine A and 1,1′-bis-valienol with porcine trehalase.** 36](#_Toc182834341)

[**Supplementary Figure 12. Phylogenetic analysis of ValL.** 37](#_Toc182834342)

[**Supplementary Figure 13. Metabolic engineering strategies for substrate supply show limited effect on the production of validamycin A and validenomycin.** 38](#_Toc182834343)

[**Reference** 40](#_Toc182834344)

**SUPPLEMENTARY TABLES**

**Supplementary Table 1 Strains used in this study and their properties and sources**

| Strains | Features | Sources |
| --- | --- | --- |
| ***Escherichia coli*** |  |  |
| DH10B | F^-^ *mcrA* ∆(mmr-*hsdRMS*-*mcrBC*) φ80*lacZ*∆M15 ∆*lac*X74 *rec*A1 *end*A1 *ara*D139 ∆(*ara*, *leu*)7997 *gal*E15 *gal*K λ-*rsp*L *nup*G | ThermoFisher SCIENTIFIC |
| ET12567(pUZ8002) | F^-^ *dam*-13::Tn9 *dcm*-6 *hsd*M *hsd*R zJJ-202::Tn10 *rec*F143 *gal*K2 *gal*T22 *ara*-14 *lac*Y1 *xyl*-5 *leu*B6 *thi*-1 *ton*A31 *rps*L136 *his*G4 tsx-78 *mtl*-1 *gln*V44 Cml^R^ Kan^R^ | [1] |
| BL21(DE3) | F^-^ *ompT hsdS_B_* (r_B_^-^ m_B_^-^) *gal* *dcm* (DE3) | Sangon Biotech |
| ***Streptomyces hygroscopicus*** **var. *jinggangensis*** | |  |
| TL01 | High-yield validamycin producing strain | [2] |
| GZY-5 | TL01Δ*valL* | This study |
| GZY-6 | TL01Δ*valG* | This study |
| GZY-11 | TL01Δ*valL*::pLQ1820 | This study |
| GZY-12 | TL01Δ*valG*::pLQ1821 | This study |

**Supplementary Table 2 Plasmids used in this study and their properties and sources**

| Plasmids | Features | Sources |
| --- | --- | --- |
| pJTU1278 | *bla, tsr, lacZ, oriT, ori^pIJ101^, ori ^ColE1^* | [3] |
| pPM927 | *bla, tsr, aadA, int^pSAM2^, oriT, ori^pBR322^* | [4] |
| pET30a (+) | *ori^pBR322^*, P_T7_, His_6-_Tag, S-Tag, Km^R^ | Takara |
| pLQ1814 | pJTU1278-derived plasmid for the deletion of *valL* in *Streptomyces hygroscopicus* | This study |
| pLQ1815 | pJTU1278-derived plasmid for the deletion of *valG* in *Streptomyces hygroscopicus* | This study |
| pLQ1816 | pET30a-derived plasmid for the expression of ValB in BL21(DE3) | This study |
| pLQ1817 | pET30a-derived plasmid for the expression of PgmA in BL21(DE3) | This study |
| pLQ1818 | pET30a-derived plasmid for the expression of ValL in BL21(DE3) | This study |
| pLQ1819 | pET30a-derived plasmid for the expression of ValO in BL21(DE3) | This study |
| pLQ1820 | pPM927-derived plasmid for the expression of *valL* under the control of *kasO*p^*^ | This study |
| pLQ1821 | pPM927-derived plasmid for the expression of *valG* under the control of *kasO*p^*^ | This study |
| pLQ1822 | pPM927-derived plasmid for the expression of *valM* under the control of *kasO*p^*^ | This study |
| pLQ1823 | pPM927-derived plasmid for the expression of *valN* under the control of *kasO*p^*^ | This study |

**Supplementary Table 3 Primers used in strain constructions**

| Primers ^a^ | Sequences (5′–3′) ^b^ |
| --- | --- |
| valB-F | ttaagaaggagatatacatatgatggacggagtgcgtgccgt |
| valB-R | ctcgagtgcggccgcaagcttcagcgccacctcttcccgct |
| pgmA-F | ctgatatcggatccgaattcatggcgcacgagcgcgcc |
| pgmA-R | tcgagtgcggccgcaagctttcagccgccgagcgctccca |
| valL-F | ctgatatcggatccgaattcgtgaccggatctgagatctt |
| valL-R | tcgagtgcggccgcaagctttcagaggtctgctcgtgtc |
| valO-F | ctgatatcggatccgaattcgtgacctgccgggtggggct |
| valO-R | tcgagtgcggccgcaagctttcacgtcagcttccctttct |
| ValL-1-F | ccgGAATTCggatttcttactgggtggcg |
| ValL-1-R | cccAAGCTTtggcgatccacgagatgttga |
| ValL-2-F | cccAAGCTTcaaccgctgagcgcttcgaca |
| ValL-2-R | ccgggtacctgcagggcgtgccgcagcag |
| ValL-yz-F | ttccggccctcaacccaagat |
| ValL-yz-R | ttcgtcgttgccatagagcgc |
| ValG-1-F | ccgGAATTCgttggcttcaactaccgtcgg |
| ValG-1-R | cccAAGCTTtgtcgaggaaggccaggagtg |
| ValG-2-F | cccAAGCTTccgtctcgacctggcatctgt |
| ValG-2-R | cccggtaccgaaccgcgcggcgaccgaac |
| ValG-yz-F | gtgtatgcgtaactcgaccct |
| ValG-yz-R | cacgatcggtcatggtgatga |
| kasOp-F | GAGGCCCTTTCGTCTTCAAGtgttcacattcgaacggtct |
| kasOp-L-R | agatccggtcatctcgagactcctgcacgctgtcgtattc |
| valL-op-F | aggagtctcgagatgaccggatctgagatctt |
| valL-op-R | CGGATGGCGGTGACGAATTctcagaggtctgctcgtgtcg |
| kasOp-G-R | cgcaccgggcatctcgagactcctgcacgctgtcgtattc |
| valG-op-F | gtctcgagatgcccggtgcgacatccca |
| valG-op-R | CGGATGGCGGTGACGAATTctcagtcaccgcgaagagacg |
| 927-YZ-F | AGTGCCACCTGACGTCTAAG |
| kasOp-M-R | cgtcgttgccatctcgagactcctgcacgctgtcgtattc |
| valM-op-F | aggagtctcgagatggcaacgacgaaacgcct |
| valM-op-R | CGGATGGCGGTGACGAATTctcacgcgatgccctccagcg |
| kasOp-N-R | ctccagagtcatctcgagactcctgcacgctgtcgtattc |
| valN-op-F | aggagtctcgagatgactctggaggagggcgg |
| valN-op-R | CGGATGGCGGTGACGAATTctcagaagggttcggggtgga |
| ValL-YZ-R | tggacgaagagcaggatcgg |
| ValG-YZ-R | acgttgagcgtccagaacca |
| ValM-YZ-R | atggcgccttcgttggcggt |
| ValN-YZ-R | tgccaccgtagagcagcacc |

^a^ F indicates forward primer, and R indicates reverse primer.

^b^ Underlined nucleotides indicate homologous sequences used for cloning with pET30a (+) or pPM927.

**Supplementary Table 4** **Medium** **components**

| Media | Components (*w/v*) ^a^ |
| --- | --- |
| Luria-Bertani broth | 0.5% yeast extract, 1% tryptone, 1% NaCl, pH 7.2 |
| SFM medium | 2% soya flour, 2% mannose, 2% agar, pH 7.2 |
| TSBY medium | 10.3% sucrose, 3% tryptone soya broth, 0.5% yeast extract, pH 7.2 |
| Fermentation medium | 9.5% rice powder, 1.8% peanut meal, 0.07% KH_2_PO_4_, 0.14% NaCl, and 0.06% CaCO_3_, pH 7.2 |
| WA medium | 2% agar, pH 7.0 |
| 2×YT medium | 1% casein hydrolysate, 1% yeast extract |

^a^ Tryptone, tryptone soya broth, yeast extract and casein hydrolysate were purchased from Oxoid. Agar, sucrose, NaCl, KH_2_PO_4_, CaCO_3_ were purchased from Sinopharm Chemical Reagent Co..

**Supplementary Table 5 HR-MS data of investigated compounds in negative ionization mode**

| Numbers | Compounds | Molecular formula | Calculated *m/z* | Detected *m/z* | Error (ppm) |
| --- | --- | --- | --- | --- | --- |
| **6** | Validenomycin | C_20_H_32_O_14_ | 495.1719 [M-H]^-^ | 495.1718 [M-H]^-^ | -0.20 |
| **6** | Validenomycin | C_20_H_32_O_14_ | 531.1486 [M+Cl]^-^ | 531.1484 [M+Cl]^-^ | -0.38 |
| **6** | Validenomycin | C_20_H_32_O_14_ | 541.1774 [M+COOH]^-^ | 541.1778 [M+COOH]^-^ | 0.74 |
| **7** | Validomycin | C_20_H_34_O_14_ | 497.1876 [M-H]^-^ | 197.1880 [M-H]^-^ | 0.80 |
| **7** | Validomycin | C_20_H_34_O_14_ | 533.1643 [M+Cl]^-^ | 533.1644 [M+Cl]^-^ | 0.20 |
| **7** | Validomycin | C_20_H_34_O_14_ | 543.1931 [M+COOH]^-^ | 543.1932 [M+COOH]^-^ | 0.18 |
| **8** | 1,1′-bis-Valienol | C_14_H_22_O_9_ | 333.1191 [M-H]^-^ | 333.1194 [M-H]^-^ | 0.90 |
| **8** | 1,1′-bis-Valienol | C_14_H_22_O_9_ | 369.0958 [M+Cl]^-^ | 369.0960 [M+Cl]^-^ | 0.54 |
| **8** | 1,1′-bis-Valienol | C_14_H_22_O_9_ | 379.1246 [M+COOH]^-^ | 379.1249 [M+COOH]^-^ | 0.79 |
| **2** | GDP-Valienol | C_17_H_25_N_5_O_15_P_2_ | 600.0750 [M-H]^-^ | 600.0748 [M-H]^-^ | -0.33 |
| **10** | 1,1′-bis-Valienol-7-phosphate | C_14_H_23_O_12_P | 413.0854 [M-H]^-^ | 413.0863 [M-H]^-^ | 2.18 |

**Supplementary Table 6 HR-MS/MS data of investigated compounds**

| Compounds | Molecular formula | Calculated  *m/z* [M-H]^-^ | Detected  *m/z* [M-H]^-^ | Error  (ppm) |
| --- | --- | --- | --- | --- |
| 1,1′-bis-Valienol (**8**) X_1_ | C_7_H_10_O_4_· | 157.0506 | 157.0507 | 0.64 |
| 1,1′-bis-Valienol (**8**) X_2_ | C_7_H_12_O_5_· | 175.0613 | 175.0617 | 2.83 |
| GDP-Valienol (**2**) X_1_ | C_10_H_15_N_5_O_11_P_2_· | 442.0171 | 442.0178 | 1.58 |
| GDP-Valienol (**2**) X_2_ | C_10_H_14_N_5_O_8_P· | 362.0507 | 362.0511 | 1.10 |
| 1,1′-bis-Valienol -7-P (**10**) X_2_ | C_7_H_11_O_7_P· | 237.0170 | 237.0171 | 0.42 |

**Supplementary Table 7 ^1^H-NMR data for validenomycin (6) (D_2_O, 600 MHz)**

| Experimental δ_H_ | Position |
| --- | --- |
| 6.13 (d, *J* = 5.1 Hz) | H6′ |
| 6.04 (d, *J* = 5.1 Hz) | H6 |
| 4.66 (d, *J* = 8.0 Hz) | H1′′ |
| 4.36 (t, *J* = 4.7 Hz) | H1′ |
| 4.31 (t, *J* = 4.7 Hz) | H1 |
| 4.30 (d, *J* = 13.9 Hz) | H7a′ |
| 4.26 (d, *J* = 7.1 Hz) | H4′ |
| 4.26 (d, *J* = 14.3 Hz) | H7a |
| 4.23 (d, *J* = 13.9 Hz) | H7b′ |
| 4.18 (d, *J* = 14.3 Hz) | H7b |
| 4.07 (d, *J* = 7.5 Hz) | H4 |
| 4.01 (dd, *J* = 10.3, 6.7 Hz) | H3′ |
| 3.93 (dd, *J* = 12.3, 1.9 Hz) | H6a′′ |
| 3.81 (dd, *J* = 10.6, 7.6 Hz) | H3 |
| 3.71-3.76 (overlapped) | H2′, H6b′′ |
| 3.66 (dd, *J* = 10.6, 4.2 Hz) | H2 |
| 3.56–3.50 (overlapped) | H3′′, H5′′ |
| 3.43 (t, *J* = 9.6 Hz) | H4′′ |
| 3.35 (t, *J* = 9.6 Hz) | H2′′ |

**Supplementary Table 8 ^13^C-NMR data for validenomycin (6) (D_2_O, 150 MHz)**

| Experimental δ_C_ | Position |
| --- | --- |
| 142.45 | C5 |
| 140.16 | C5′ |
| 123.23 | C6′ |
| 120.96 | C6 |
| 103.21 | C1′′ |
| 82.43 | C4′ |
| 75.98 | C5′′ |
| 75.61 | C3′′ |
| 73.79 | C1 |
| 73.35 | C1′ |
| 73.25 | C2′′ |
| 72.78 | C3 |
| 72.15 | C4 |
| 71.42 | C3′ |
| 70.73 | C2 |
| 70.18 | C2′ |
| 69.54 | C4′′ |
| 61.41 | C7′ |
| 61.27 | C7 |
| 60.62 | C6′′ |

**Supplementary Table 9 ^1^H-NMR data for validomycin (7) (D_2_O, 600 MHz)**

| Experimental δ_H_ | Position |
| --- | --- |
| 6.06 (d, *J* = 4.1 Hz) | H6 |
| 4.55 (d, *J* = 7.9 Hz) | H1′′ |
| 4.26 (d, *J* = 14.2 Hz) | H7a |
| 4.21 (t, *J* = 4.6 Hz) | H1 |
| 4.19 (d, *J* = 14.2 Hz) | H7b |
| 4.11 (d, *J* = 7.3 Hz) | C4 |
| 4.03 (m) | H1′ |
| 3.93 (dd, *J* = 10.2, 7.3 Hz) | H3 |
| 3.93 (dd, *J* = 12.5, 2.1 Hz) | H7a′ |
| 3.78 (t, *J* = 8.3 Hz) | H3′ |
| 3.76 (dd, *J* = 12.8, 5.5 Hz) | H6a′′ |
| 3.78 – 3.84 (overlapped) | H7a′, H7b′ |
| 3.68 (dd, *J* = 10.5, 4.1 Hz) | H2 |
| 3.47 – 3.58 (overlapped) | H3′′, H5′′ |
| 3.53 (t, *J* = 10.0 Hz) | H4′ |
| 3.51 (dd, *J* = 9.2, 8.2 Hz) | H2′ |
| 3.45 (t, *J* = 10.5 Hz) | H4′′ |
| 3.36 (dd, *J* = 9.2, 8.2 Hz) | H2′′ |
| 2.16 (dt, *J* = 14.8, 3.6 Hz) | H6′_eq_ |
| 2.13 (m) | H5′ |
| 1.37 (br t, *J* = 13.3 Hz) | H6′_ax_ |

**Supplementary Table 10 ^13^C-NMR data for validomycin (7) (D_2_O, 150 MHz)**

| Experimental δ_C_ | Position |
| --- | --- |
| 142.46 | C5 |
| 120.96 | C6 |
| 102.82 | C1′′ |
| 83.87 | C4′ |
| 75.98 | C5′′ |
| 75.58 | C3′′ |
| 74.27 | C1′ |
| 73.58 | C2′ |
| 73.40 | C2′′ |
| 73.13 | C3′ |
| 72.89 | C3 |
| 72.27 | C4 |
| 71.55 | C1 |
| 70.66 | C2 |
| 69.38 | C4′′ |
| 61.56 | C7′ |
| 61.36 | C7 |
| 60.48 | C6′′ |
| 37.54 | C5′ |
| 26.87 | C6′ |

**Supplementary Table 11 ^1^H-NMR data for 1,1′-bis-valienol (8) (D_2_O, 600 MHz)**

| Experimental δ_H_ | Position |
| --- | --- |
| 6.06 (d, *J* = 4.1 Hz) | H6, H6′ |
| 4.36 (t, *J* = 4.6 Hz) | H1, H1′ |
| 4.27 (d, *J* = 14.3 Hz) | H7a, H7a′ |
| 4.19 (d, *J* = 14.3 Hz) | H7b, H7b′ |
| 4.09 (d, *J* = 7.5 Hz) | H4, H4′ |
| 3.82 (dd, *J* = 10.5, 7.6 Hz) | H3, H3′ |
| 3.68 (dd, *J* = 10.6, 4.2 Hz) | H2, H2′ |

**Supplementary Table 12 ^13^C-NMR data for 1,1′-bis-valienol (8) (D_2_O, 150 MHz)**

| Experimental δ_C_ | Position |
| --- | --- |
| 142.40 | C5, C5′ |
| 121.01 | C6, C6′ |
| 73.52 | C1, C1′ |
| 72.80 | C3, C3′ |
| 72.13 | C4, C4′ |
| 70.70 | C2, C2′ |
| 61.29 | C7, C7′ |

**SUPPLEMENTARY FIGURES**





**Supplementary Figure 1. Chemical structures of validamycin A (1) and its derivatives involved in this study.**

**a**


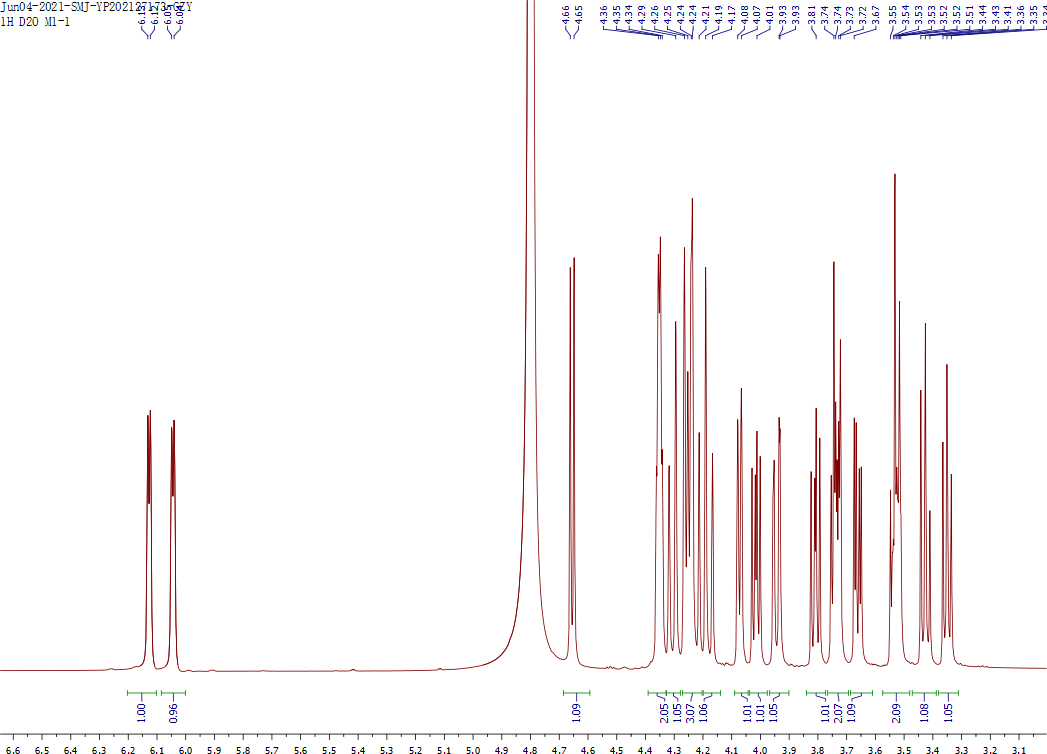


**b
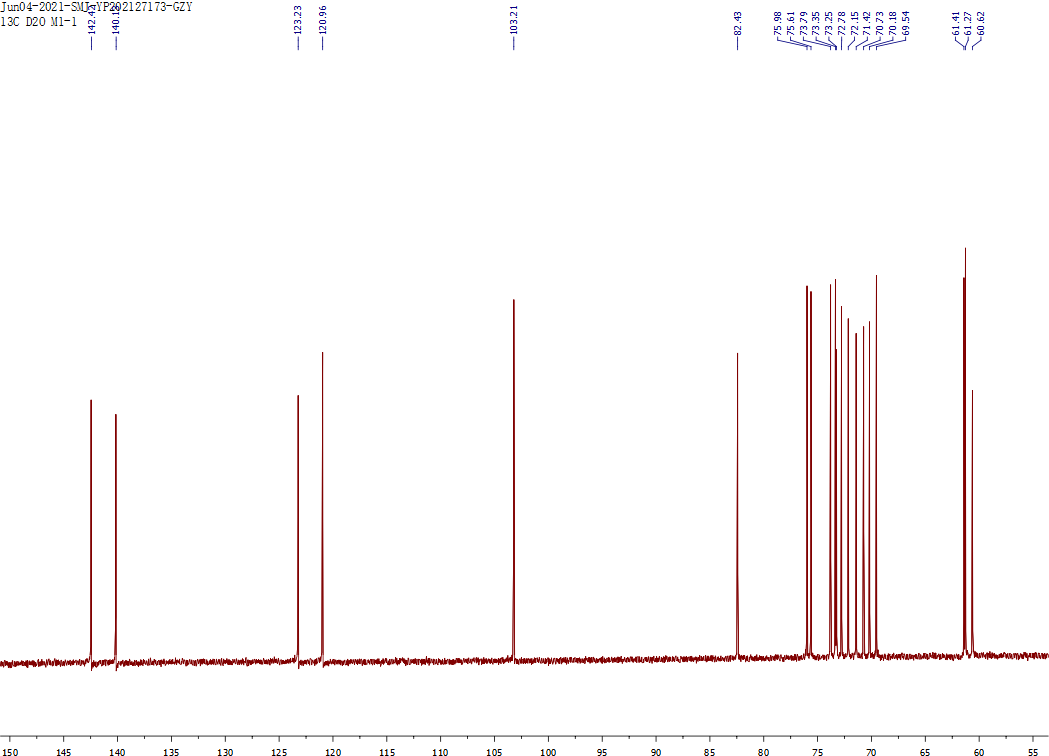
**

**c**


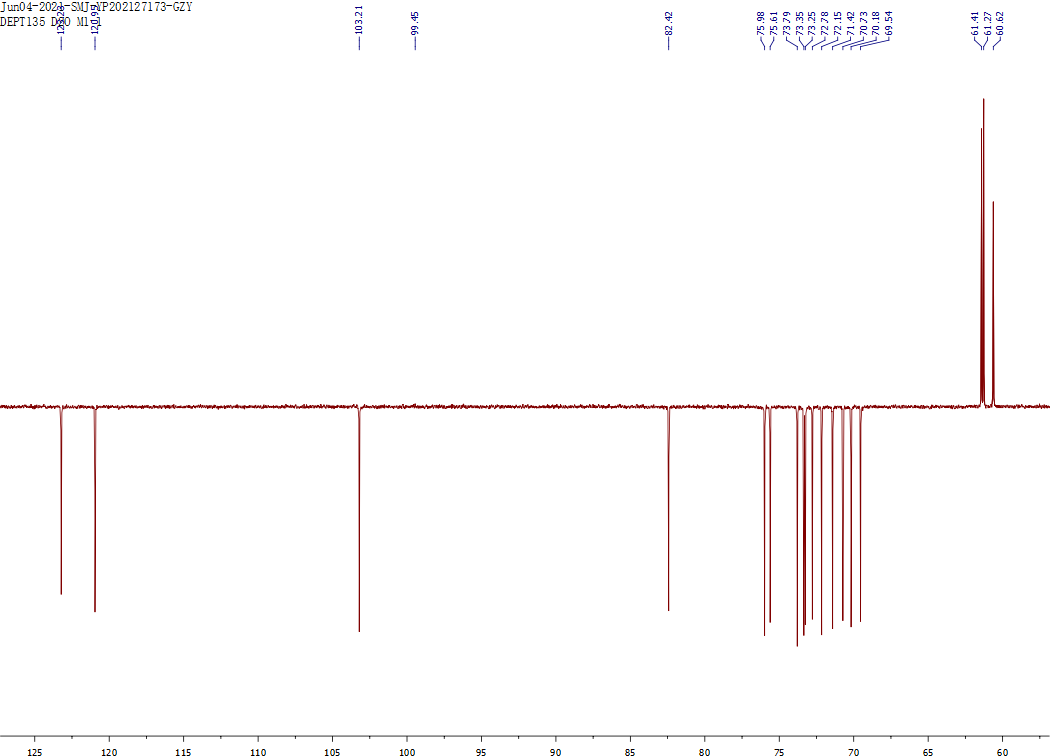


**d**


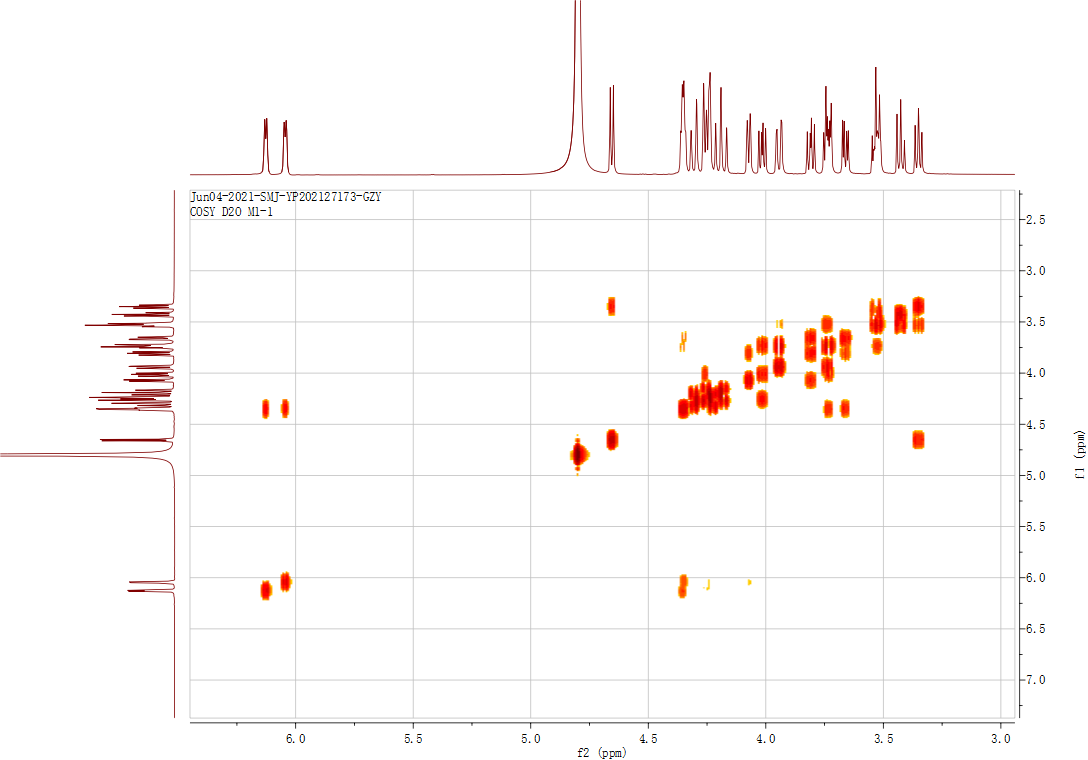


**e**


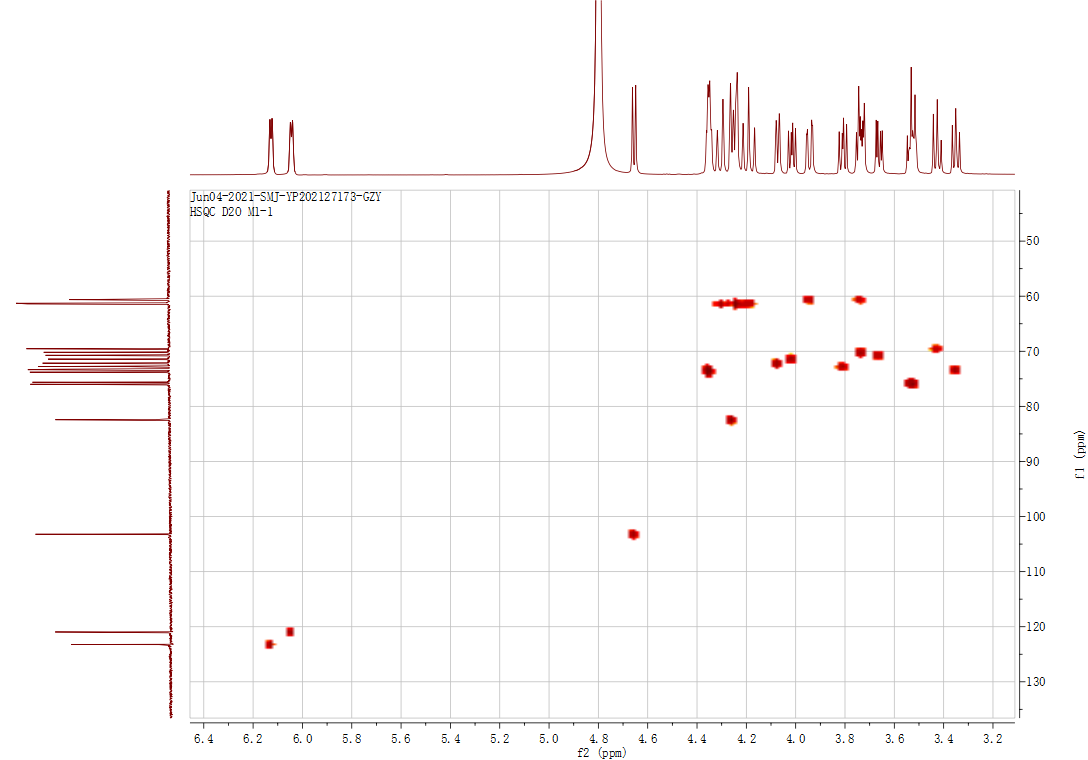


**f**


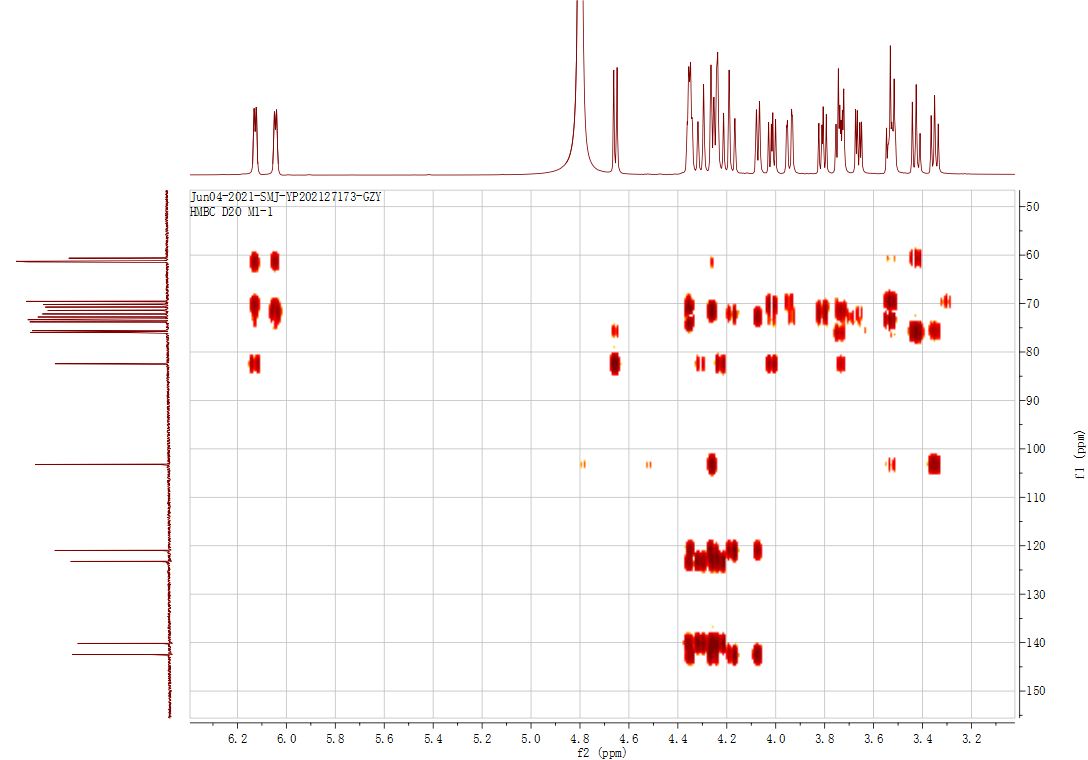


**g**
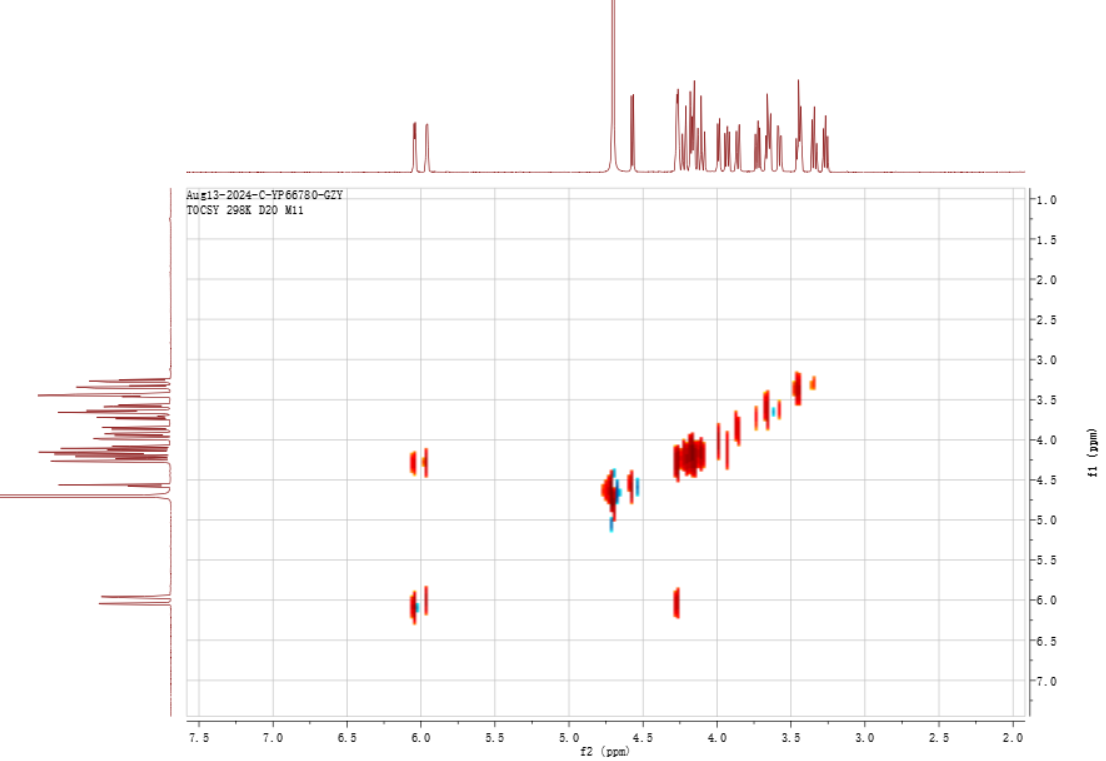


**h**

**Supplementary Figure 2. NMR analysis of validenomycin (6).**

**a,** ^1^H-NMR. **b**, ^13^C-NMR. **c**, DEPT NMR. **d**, COSY NMR. **e**, HSQC NMR. **f**, HMBC NMR. **g**, TOCSY NMR. **h**, Chemical structure of validenomycin (**6**).

**a**
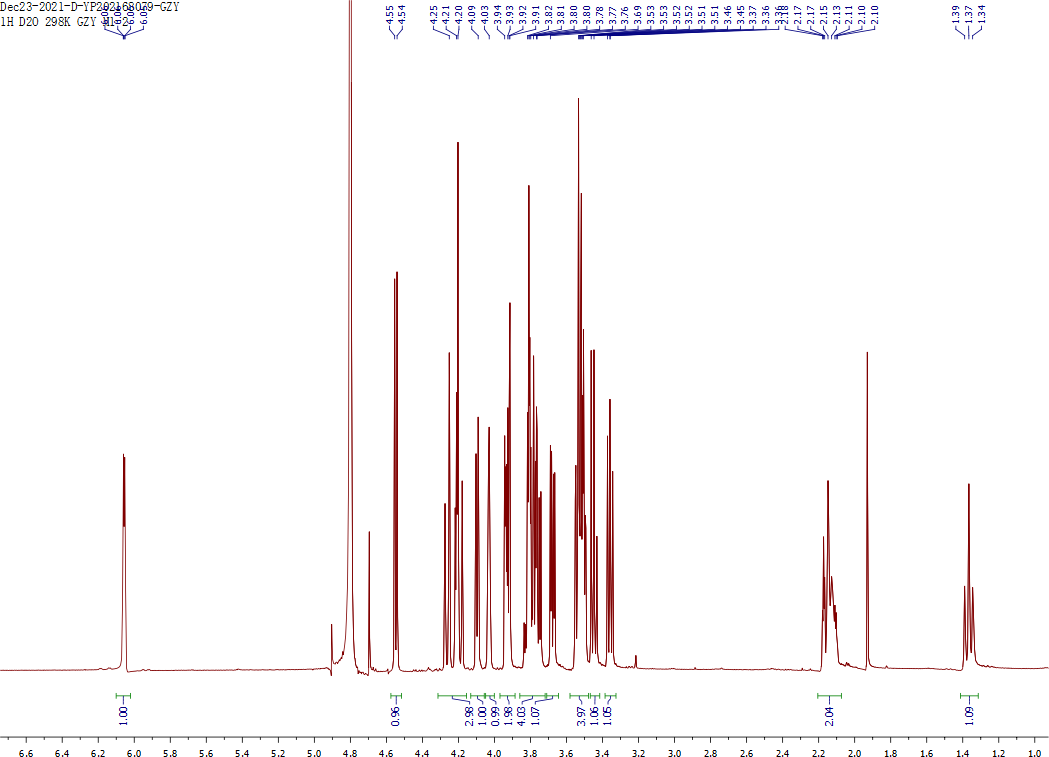


**b
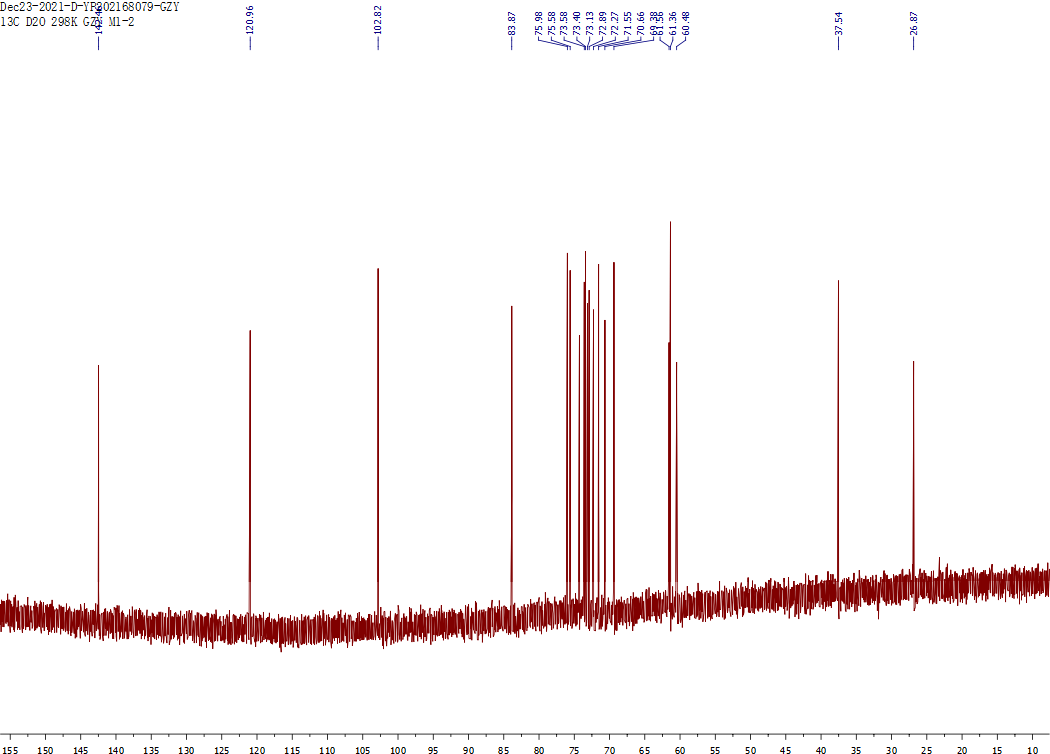
**

**c**


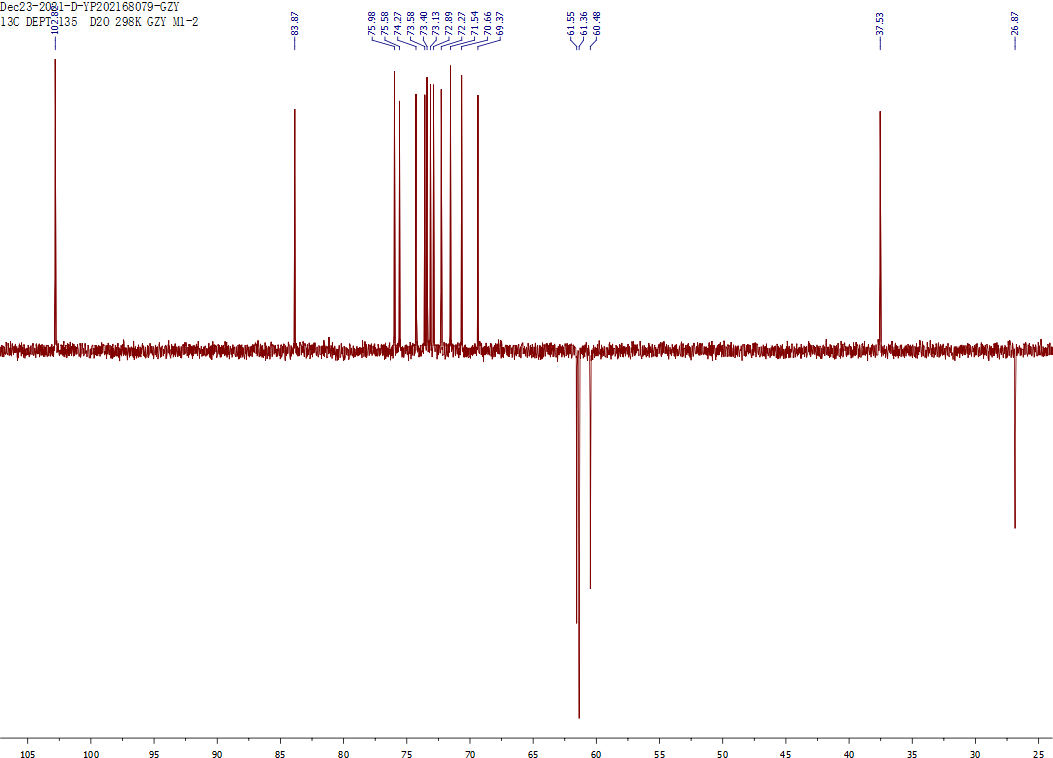


**d**
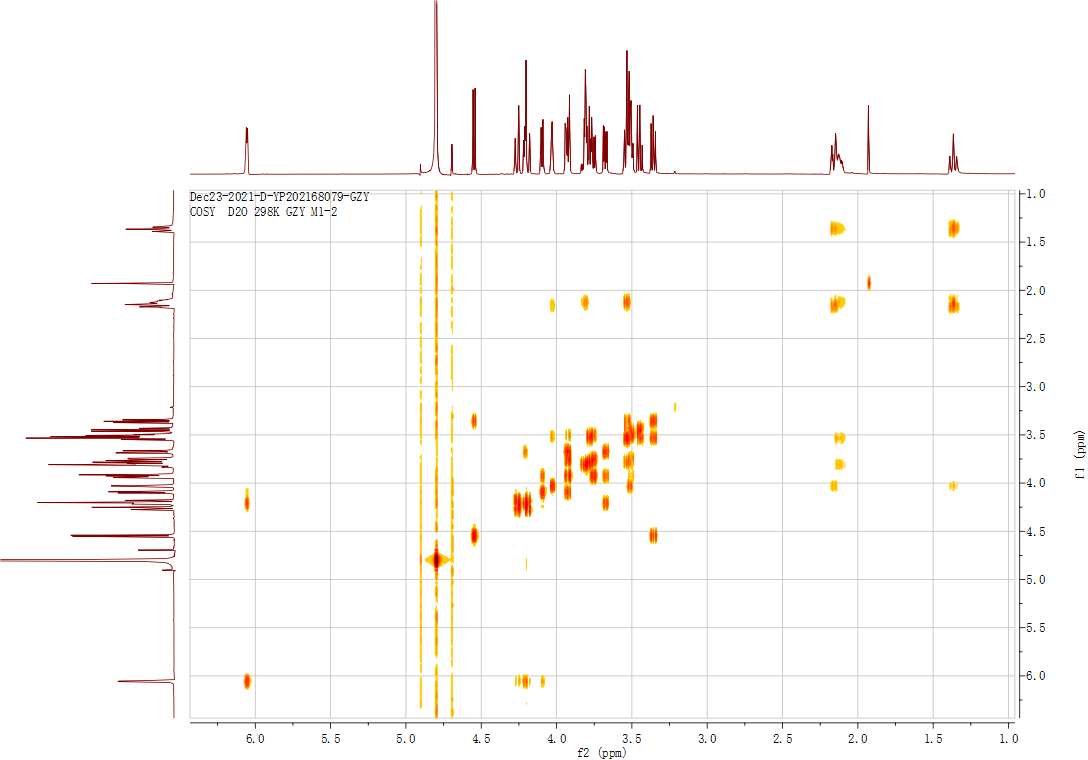


**e**


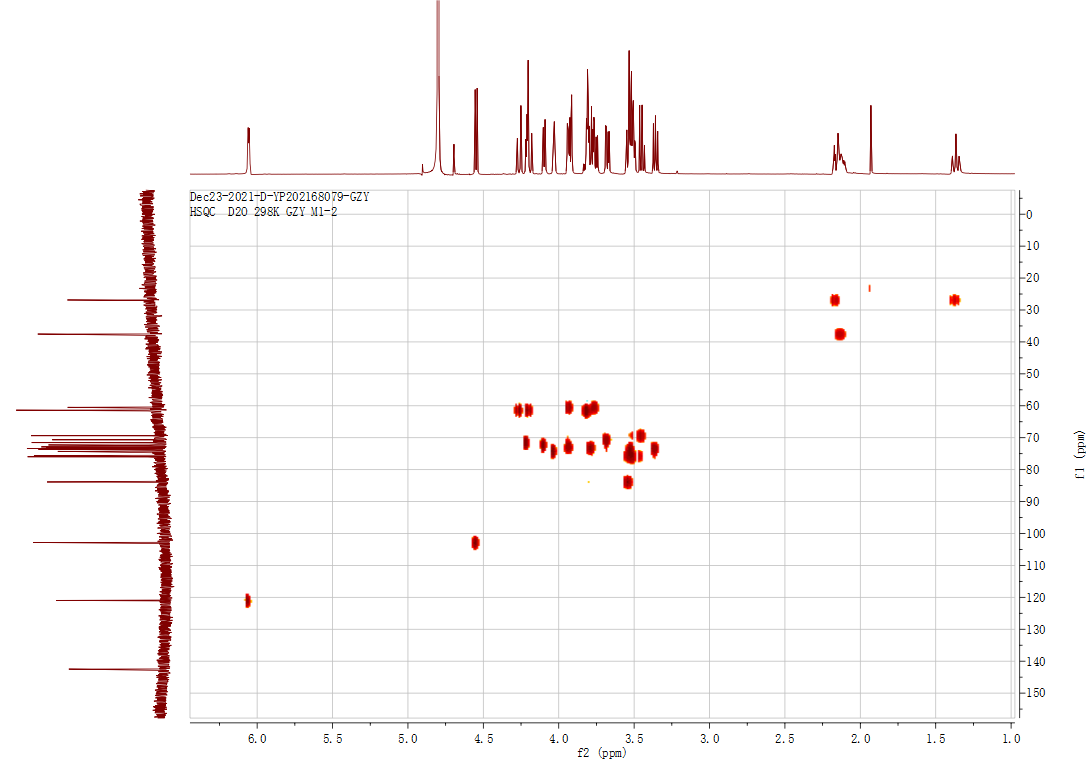


**f**


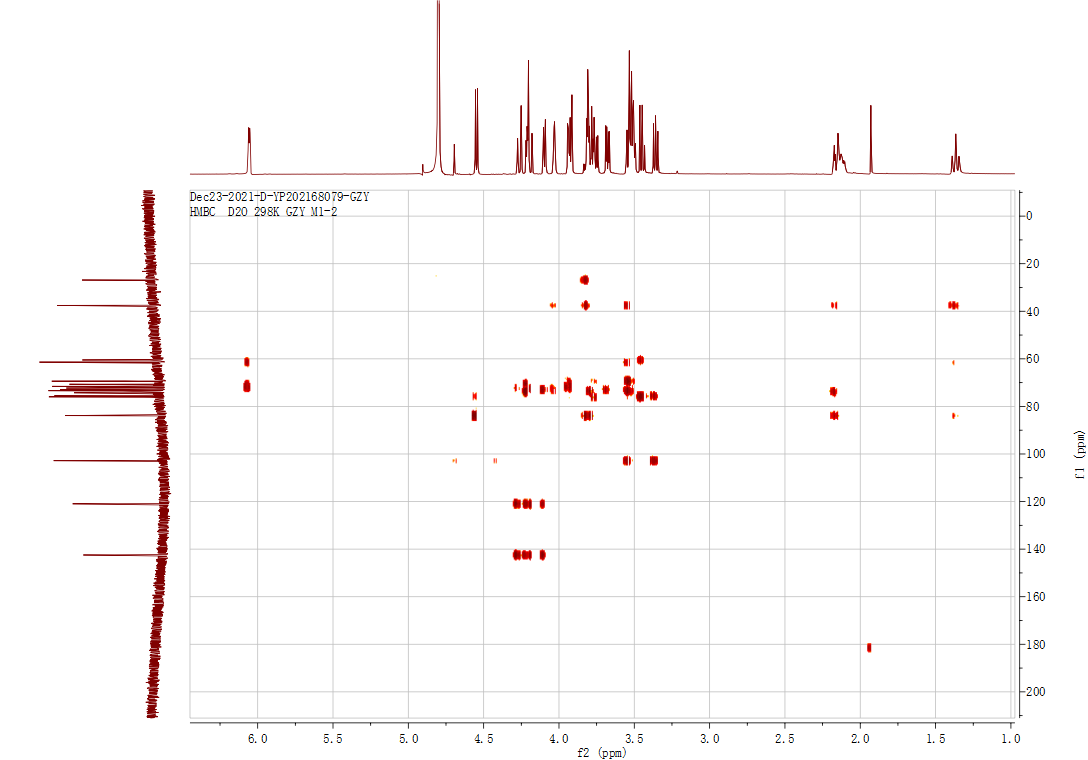


**g**
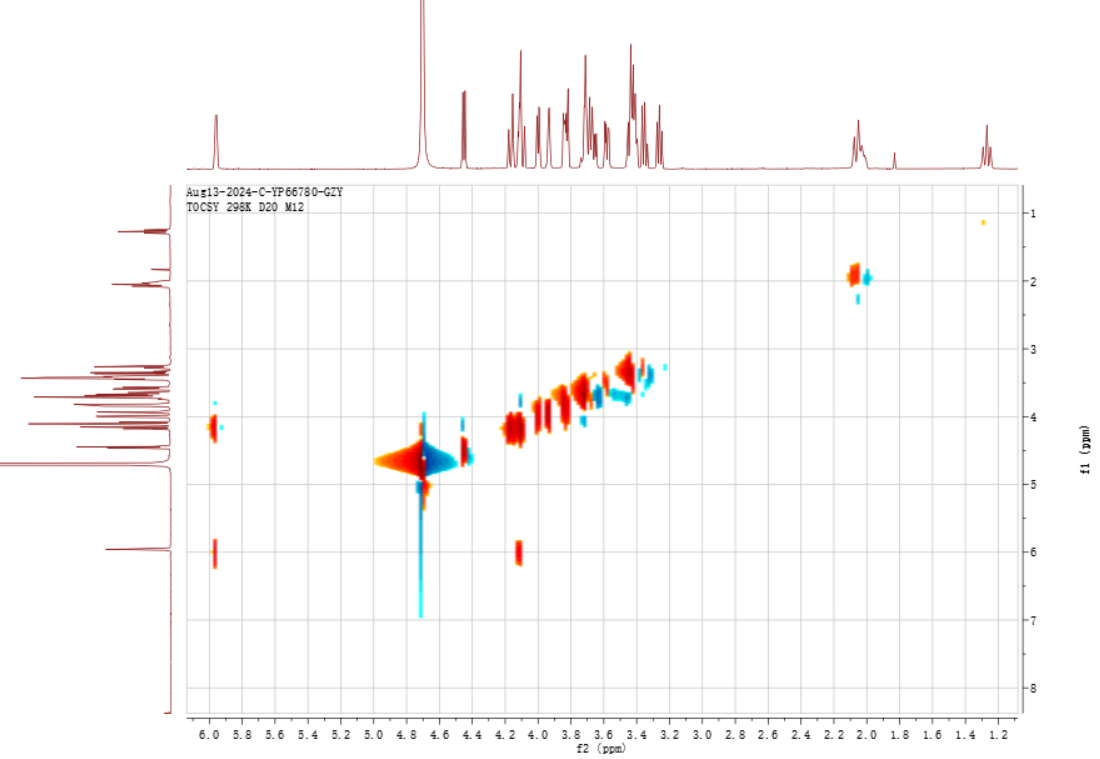


**h**

**Supplementary Figure 3. NMR analysis of validomycin (7).**

**a,** ^1^H-NMR. **b**, ^13^C-NMR. **c**, DEPT NMR. **d**, COSY NMR. **e**, HSQC NMR. **f**, HMBC NMR. **g**, TOCSY NMR. **h**, Chemical structure of validomycin (**7**).

**a
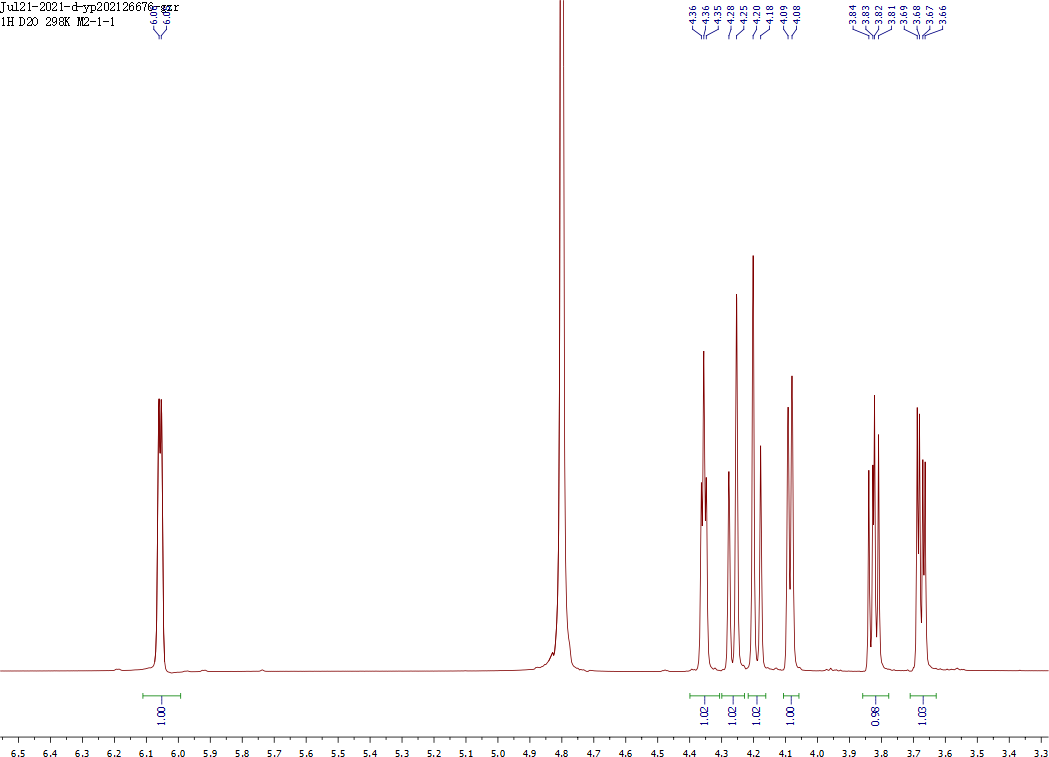
**

**b
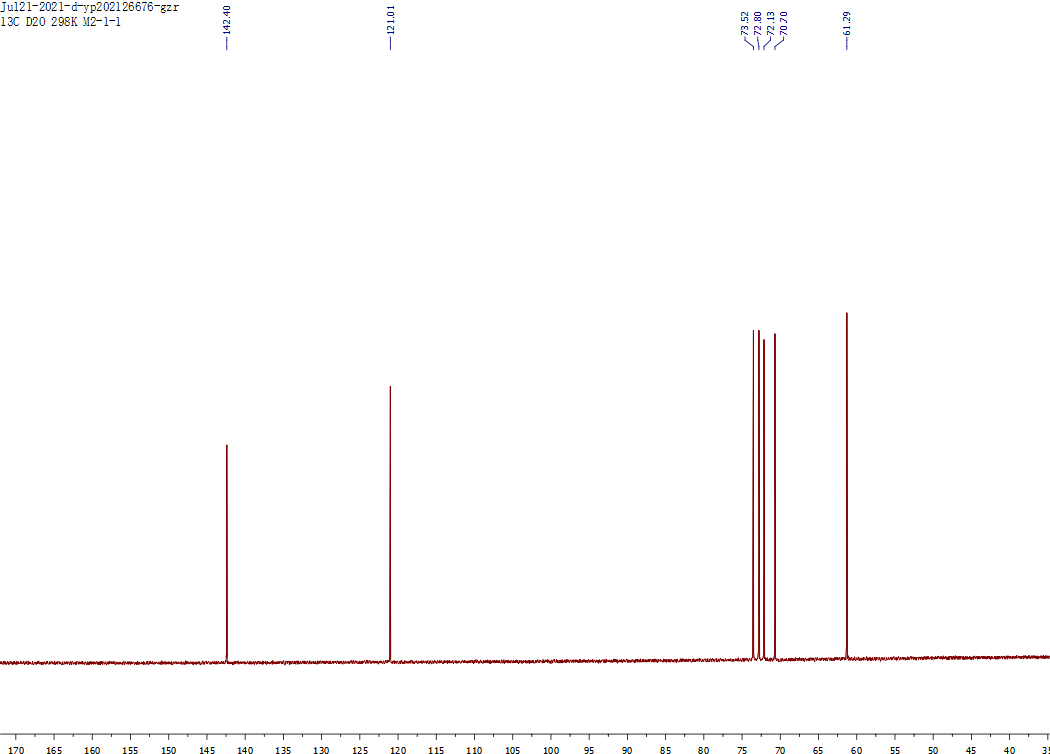
**

**c**

**
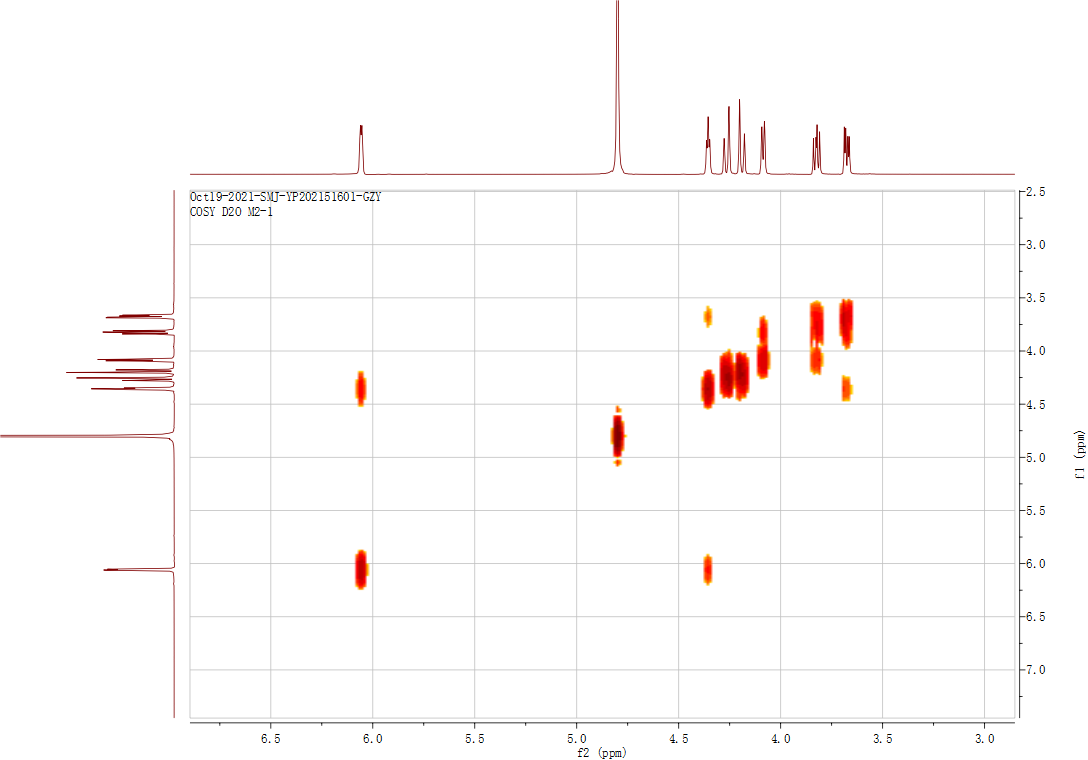
**

**d**
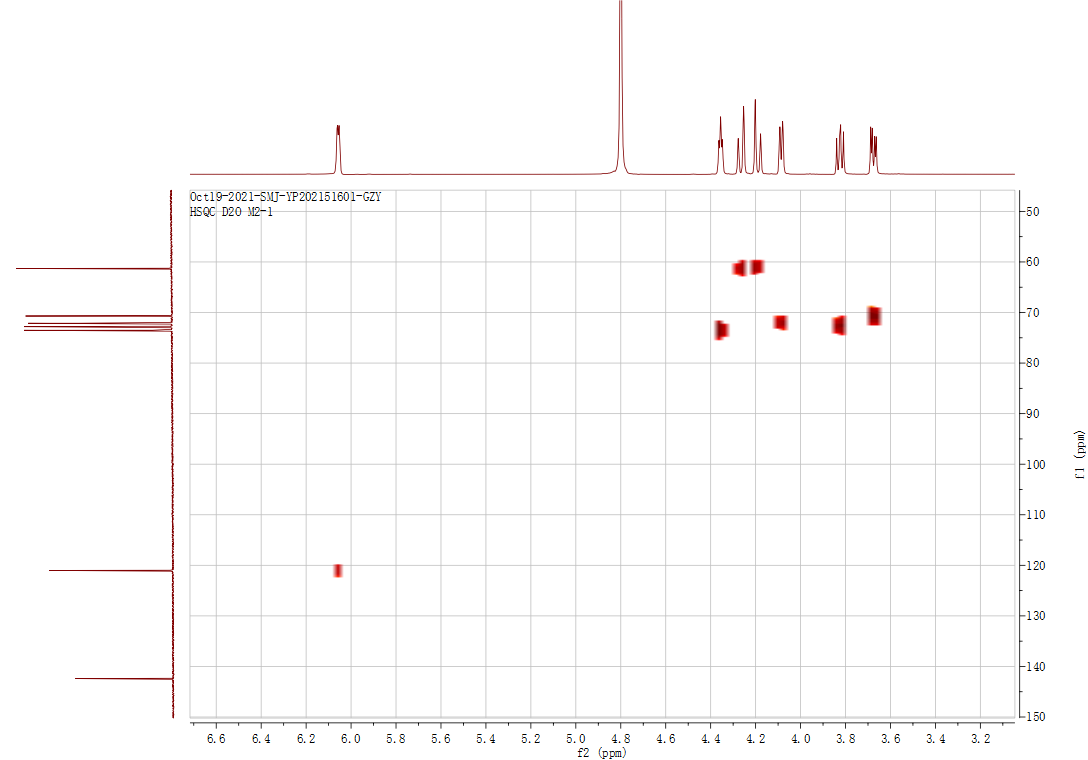


**e**


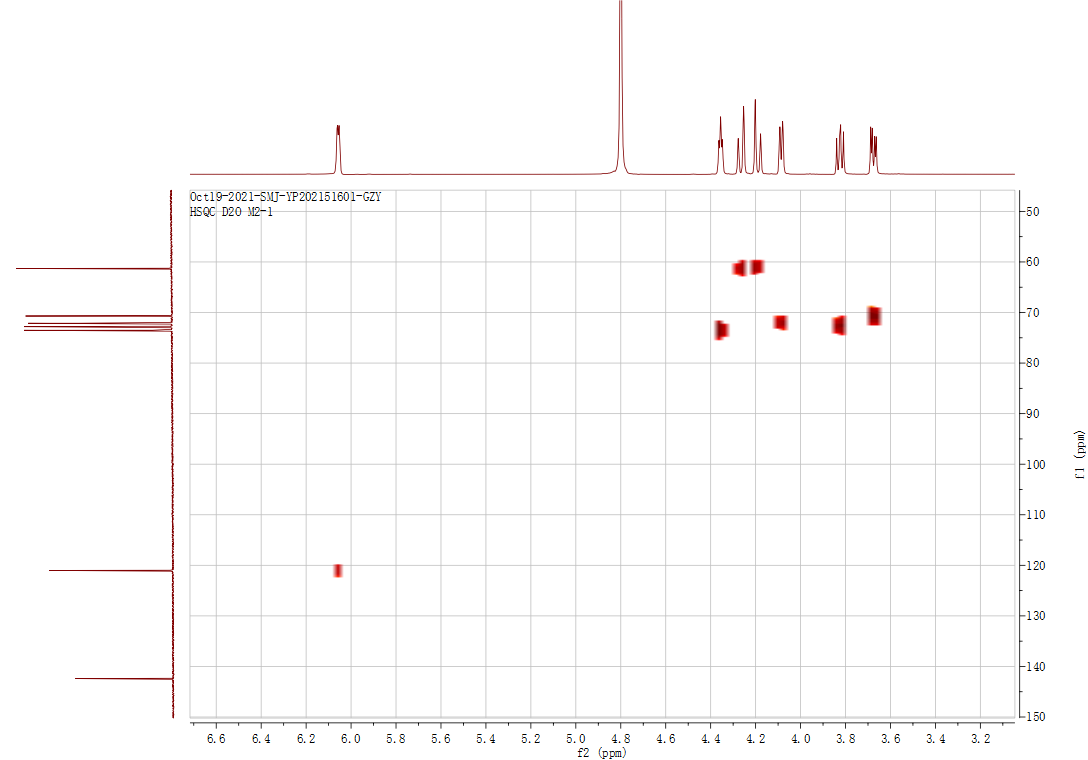


**f**


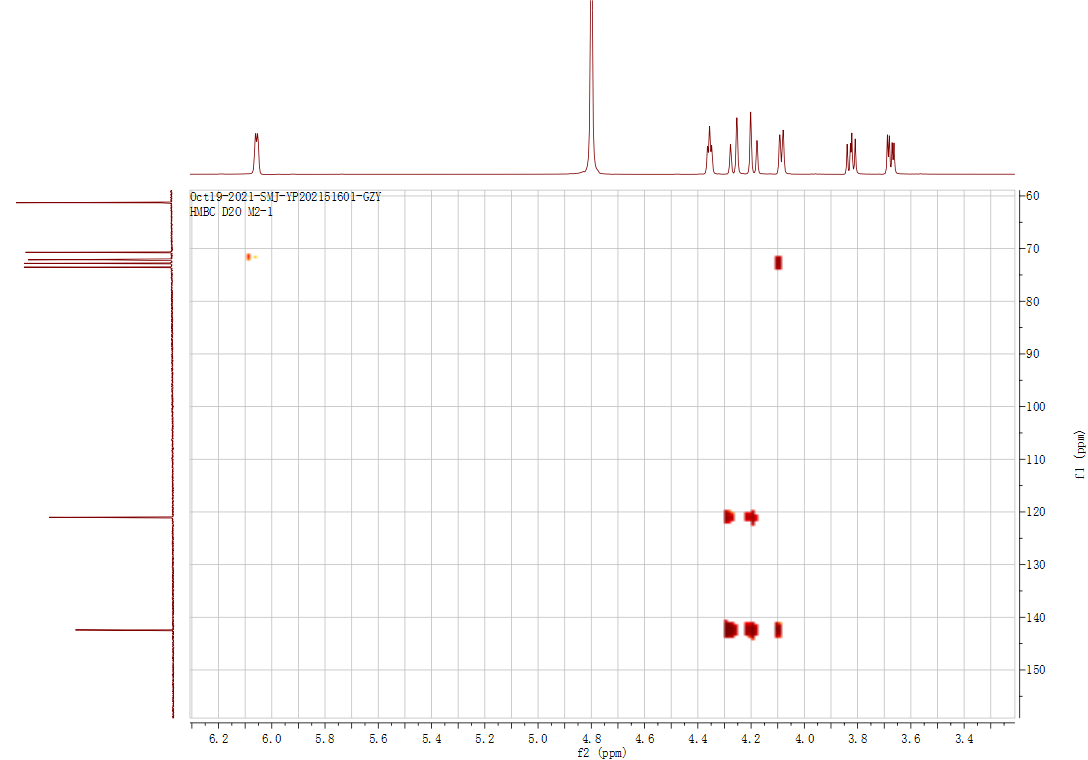


**g**
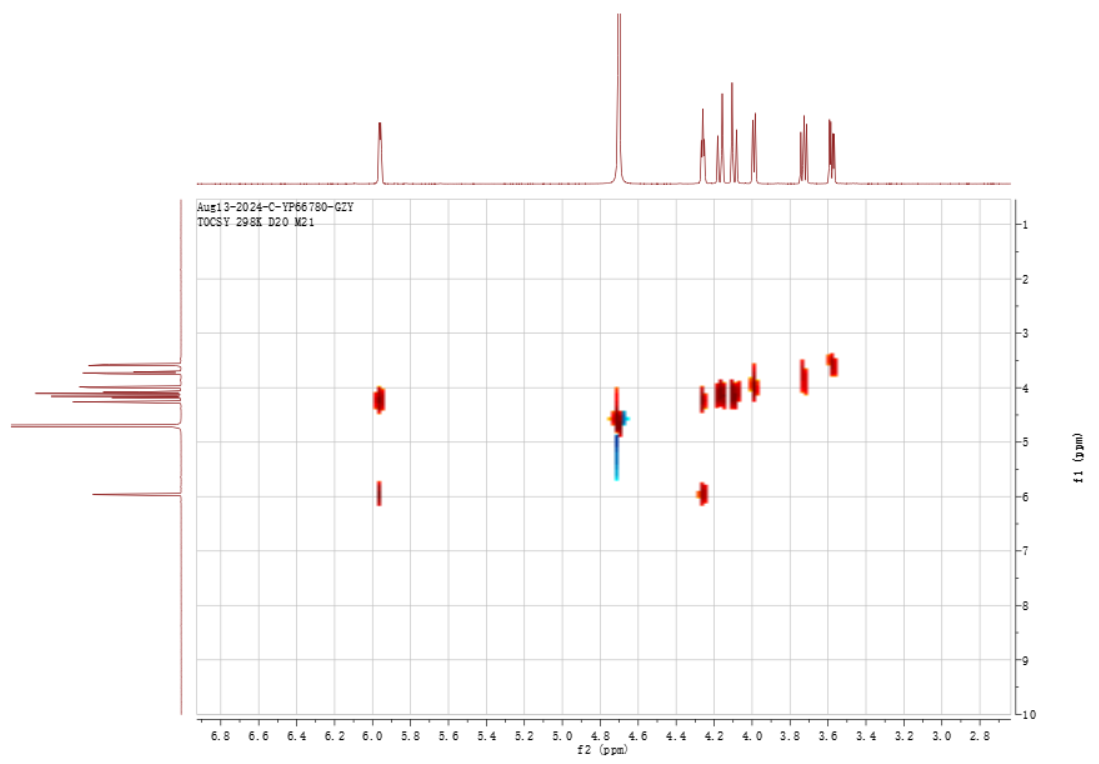


**h**

**Supplementary Figure 4. NMR analysis of 1,1′-bis-valienol (8).**

**a,** ^1^H-NMR. **b**, ^13^C-NMR. **c**, DEPT NMR. **d**, COSY NMR. **e**, HSQC NMR. **f**, HMBC NMR. **g**, TOCSY NMR. **h**, Chemical structure of 1,1′-bis-valienol (**8**).

**
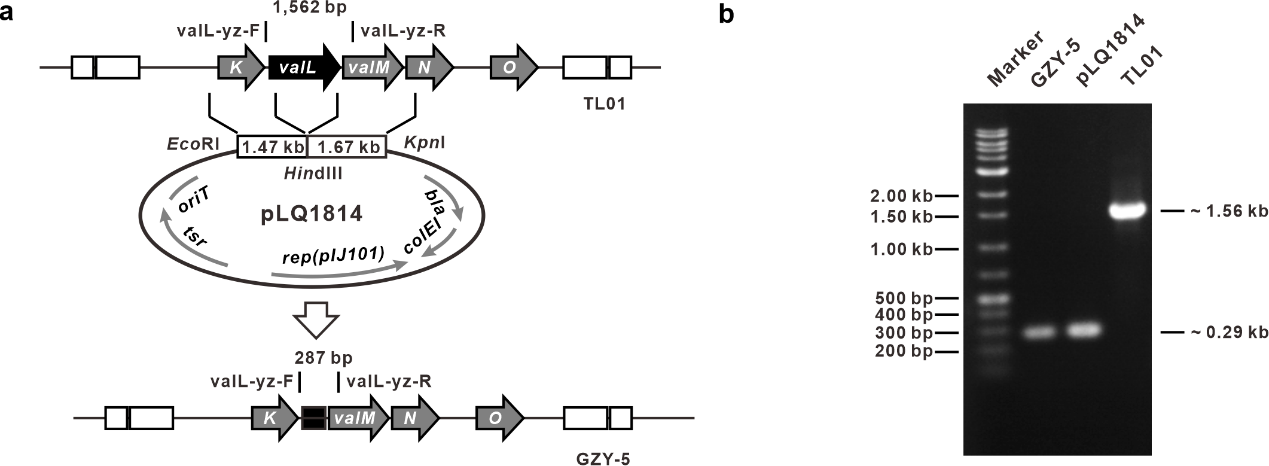
**

**Supplementary Figure 5. Construction of GZY-5 strain.**

**a**, Diagram of homologous double crossovers for *valL* deletion in *S. hygroscopicus* var. *jinggangensis* TL01. **b**, Verification of the *valL* deletion mutant GZY-5 by gel electrophoresis of PCR products; PCR was performed using valL-yz-L and valL-yz-R as primers. Lanes were as follows: M, DNA marker; GZY-5, PCR product using GZY-5 (TL01Δ*valL*) genomic DNA as template; pLQ1814, PCR product using pLQ1814 plasmid DNA as template; TL01, PCR product using strain TL01 genomic DNA as template.


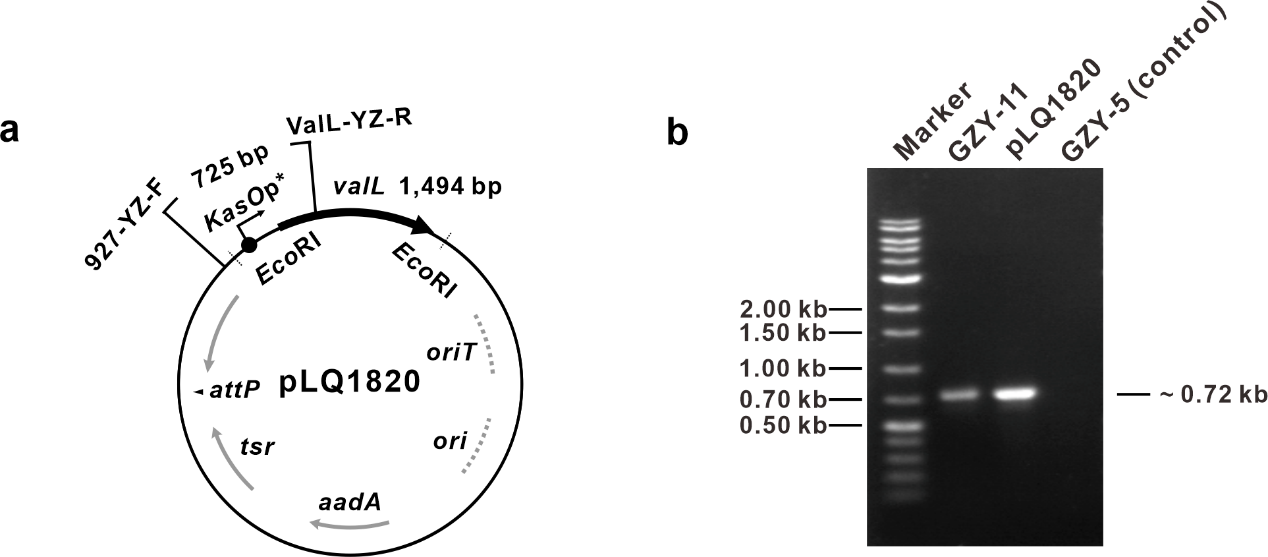


**Supplementary Figure 6. Construction of GZY-11 strain.**

**a**, Schematic of the pLQ1820 plasmid. This plasmid was constructed with pPM927 as the backbone, incorporating the gene *valL* under the control of *kasO*p*** promoter. **b**, Verification of GZY-11 strain by gel electrophoresis of PCR products. PCR was performed using 927-YZ-F and ValL-YZ-R as primers. Lanes were as follows: M, DNA marker; GZY-11, PCR product using GZY-11 (TL01Δ*valL*::pLQ1820) genomic DNA as template; pLQ1820, PCR product using pLQ1820 plasmid DNA as template; GZY-5, PCR product using GZY-5 (TL01Δ*valL*) genomic DNA as template.


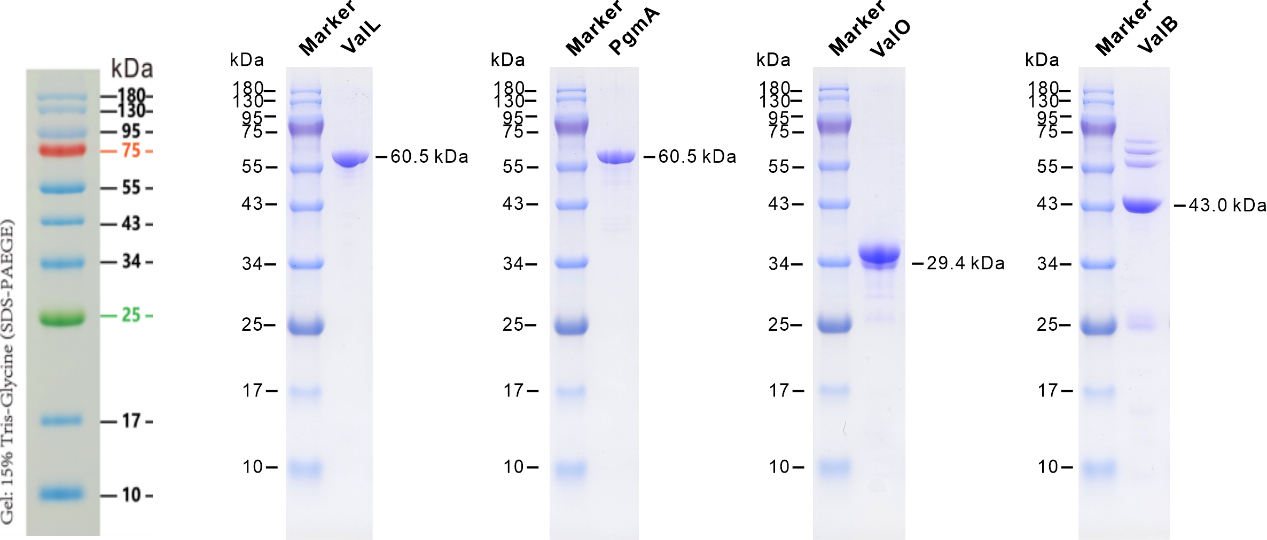


**Supplementary Figure 7. Overexpression of recombinant His-tagged target proteins in *E. coli* BL21(DE3) and analysis by SDS-PAGE.**


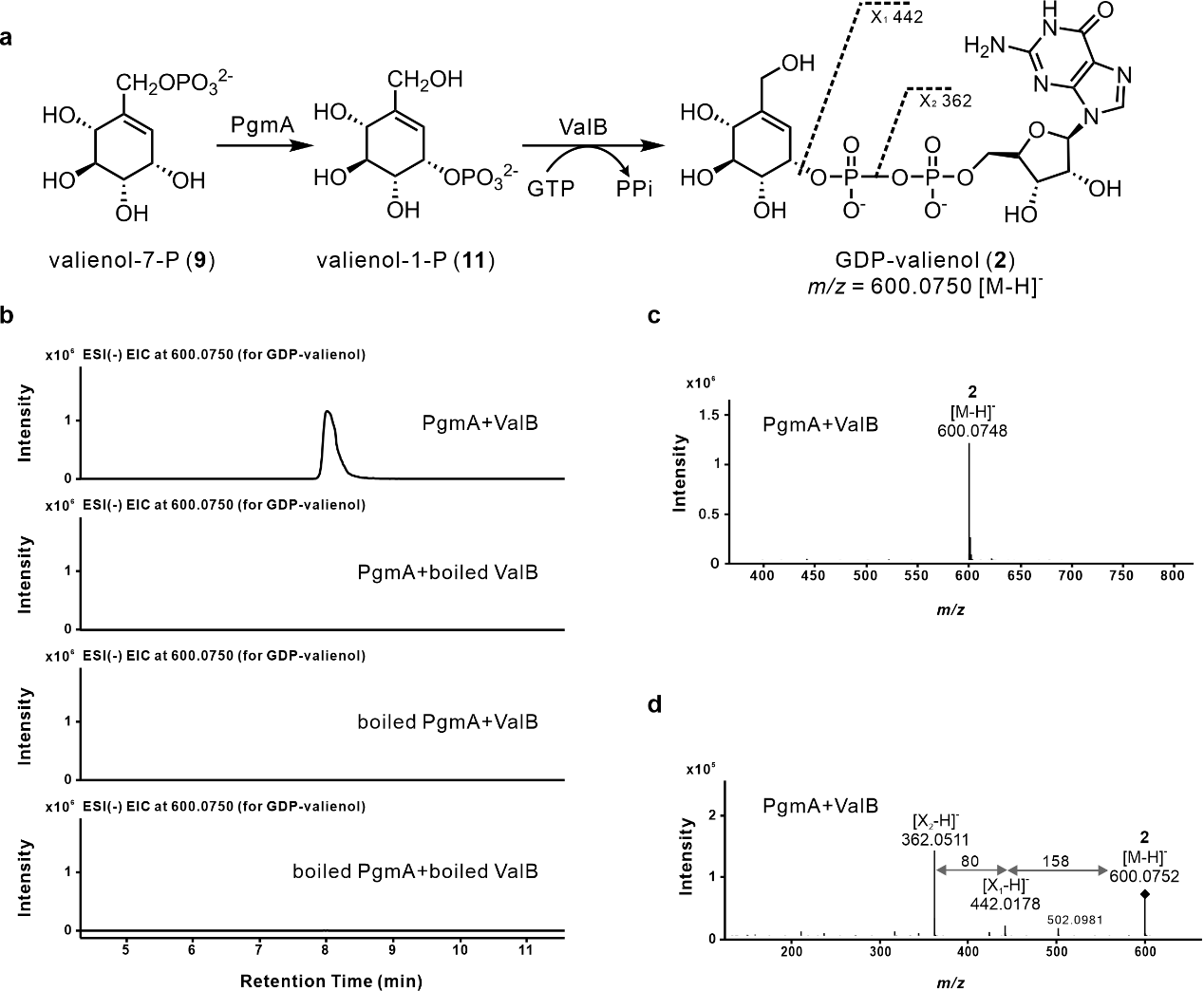


**Supplementary Figure 8. HR-MS and HR-MS/MS analysis of PgmA and ValB reaction products.**

**a**, Reaction scheme of PgmA and ValB, ValL and ValO. **b**, HPLC-HR/MS EIC chromatograms (negative ion mode) of PgmA and ValB reaction with valienol-7-phsphate (**9**) and GTP, and the negative control with boiled PgmA or ValB. All chromatograms are the extraction of the corresponding calculated exact mass for GDP-valienol (**2**) (*m/z* value of 600.0750 [M-H]^–^). **c**, HR-MS of reaction products of PgmA and ValB. **d**, HR-MS/MS of reaction products

**
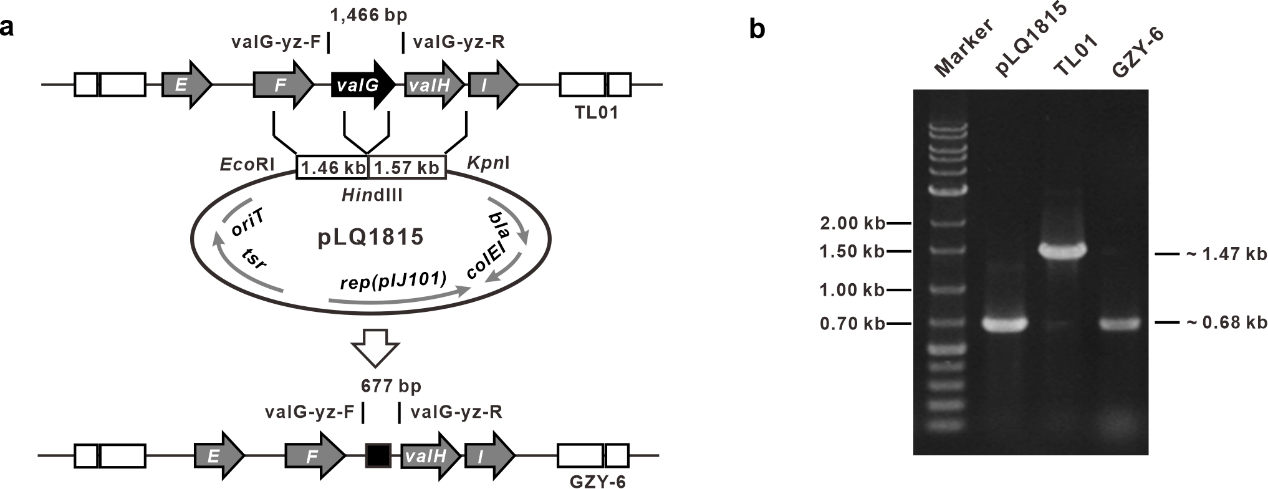
**

**Supplementary Figure 9. Construction of GZY-6 strain.**

**a**, Diagram of homologous double crossovers for *valG* deletion in *S. hygroscopicus* var. *jinggangensis* TL01. **b**, Verification of the *valG* deletion mutant GZY-6 by gel electrophoresis of PCR products; PCR was performed using valG-yz-L and valG-yz-R as primers. Lanes were as follows: M, DNA marker; pLQ1815, PCR product using pLQ1815 plasmid DNA as template; TL01, PCR product using strain TL01 genomic DNA as template; GZY-6, PCR product using GZY-6 (TL01Δ*valG*) genomic DNA as template.

**
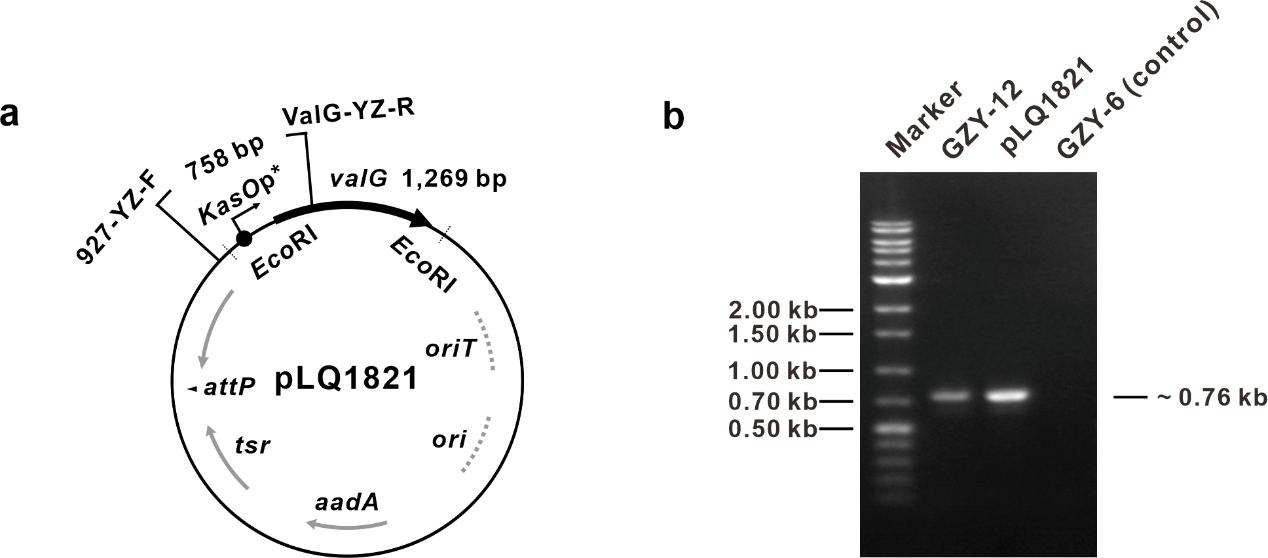
**

**Supplementary Figure 10. Construction of GZY-12 strain.**

**a**, Schematic of the pLQ1821 plasmid. This plasmid was constructed with pPM927 as the backbone, incorporating the gene *valG* under the control of *kasO*p*** promoter. **b**, Verification of GZY-12 strain by gel electrophoresis of PCR products. PCR was performed using 927-YZ-F and ValL-YZ-R as primers. Lanes were as follows: M, DNA marker; GZY-12, PCR product using GZY-12 (TL01Δ*valG*::pLQ1821) genomic DNA as template; pLQ1821, PCR product using pLQ1821 plasmid DNA as template; GZY-6, PCR product using GZY-6 (TL01Δ*valG*) genomic DNA as template.


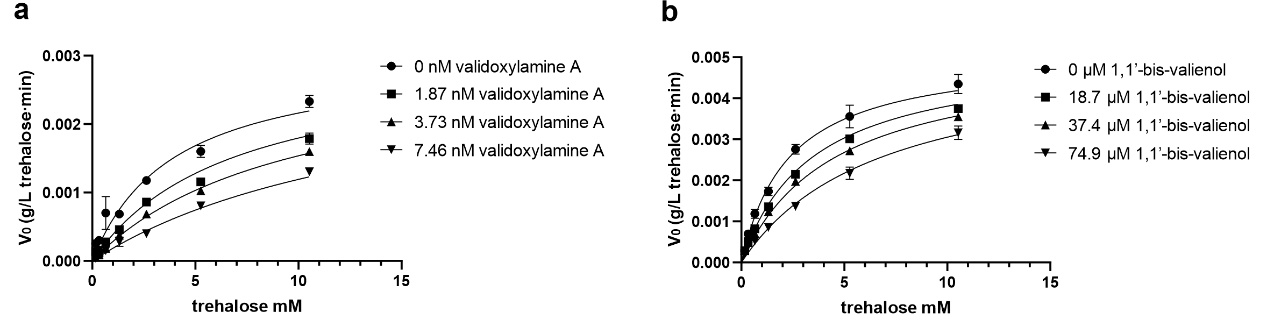


**Supplementary Figure 11. Inhibitory kinetics of validoxylamine A (5) and** **1,1′-bis-valienol (8) with porcine trehalase.**

**a**, Evaluating *Ki* value of validoxylamine A (**5**) with porcine trehalase. **b**, Evaluating *Ki* value of 1,1′-bis-valienol (**8**) with porcine trehalase.


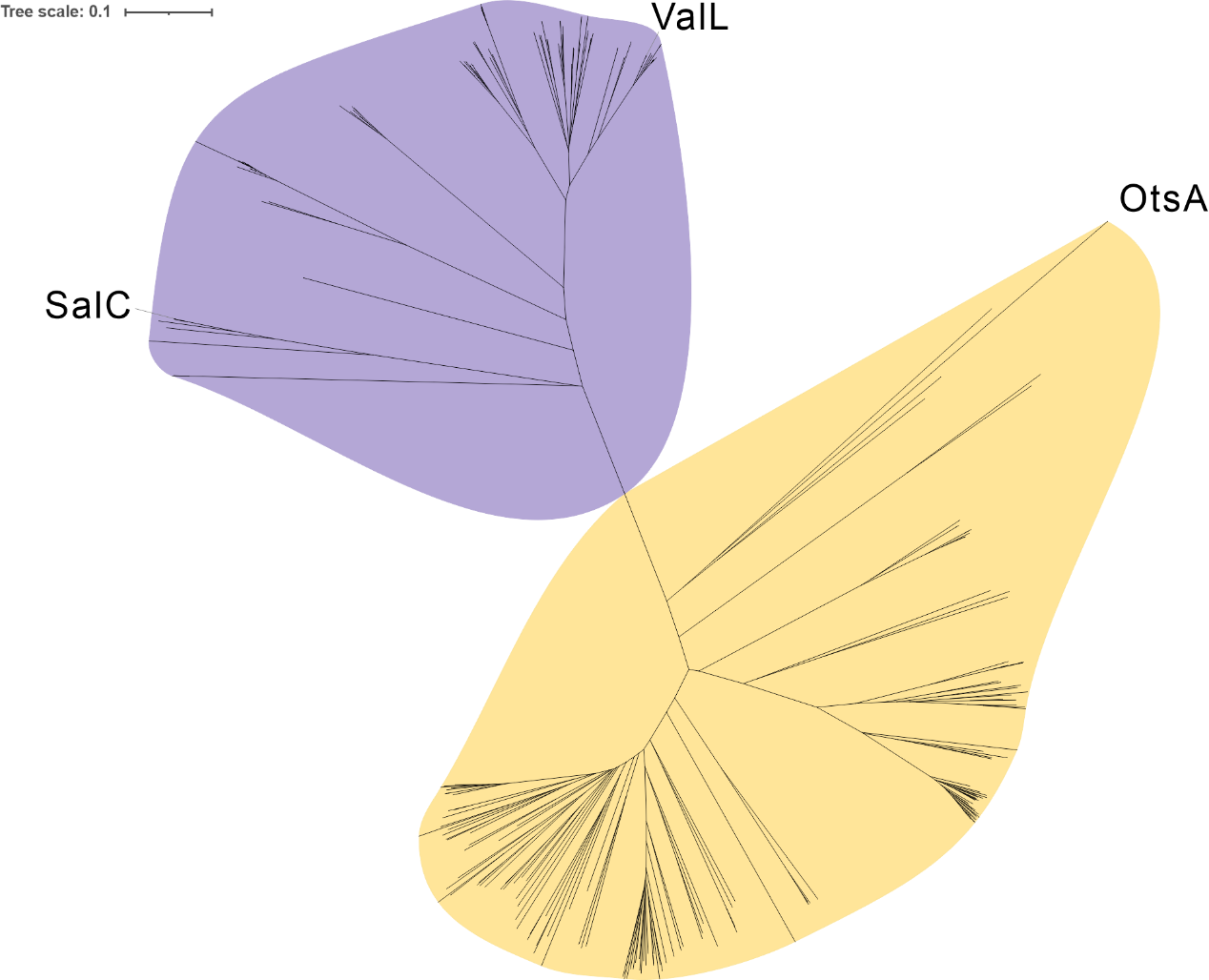


**Supplementary Figure 12. Phylogenetic analysis of ValL.**

The protein sequences of ValL and its homologous proteins were used to construct phylogenetic trees. The phylogenetic tree was constructed by Geneious Prime (version 2023.2.1) using Jukes-Cantor genetic distance model and Neighbor-Joining tree building method. The constructed tree was edited by iTOL[5]. The ones shaded in purple are predicted to be in the aminocyclitol biosynthetic gene clusters. The ones shaded in yellow are predicted to be not in the aminocyclitol biosynthetic gene clusters.


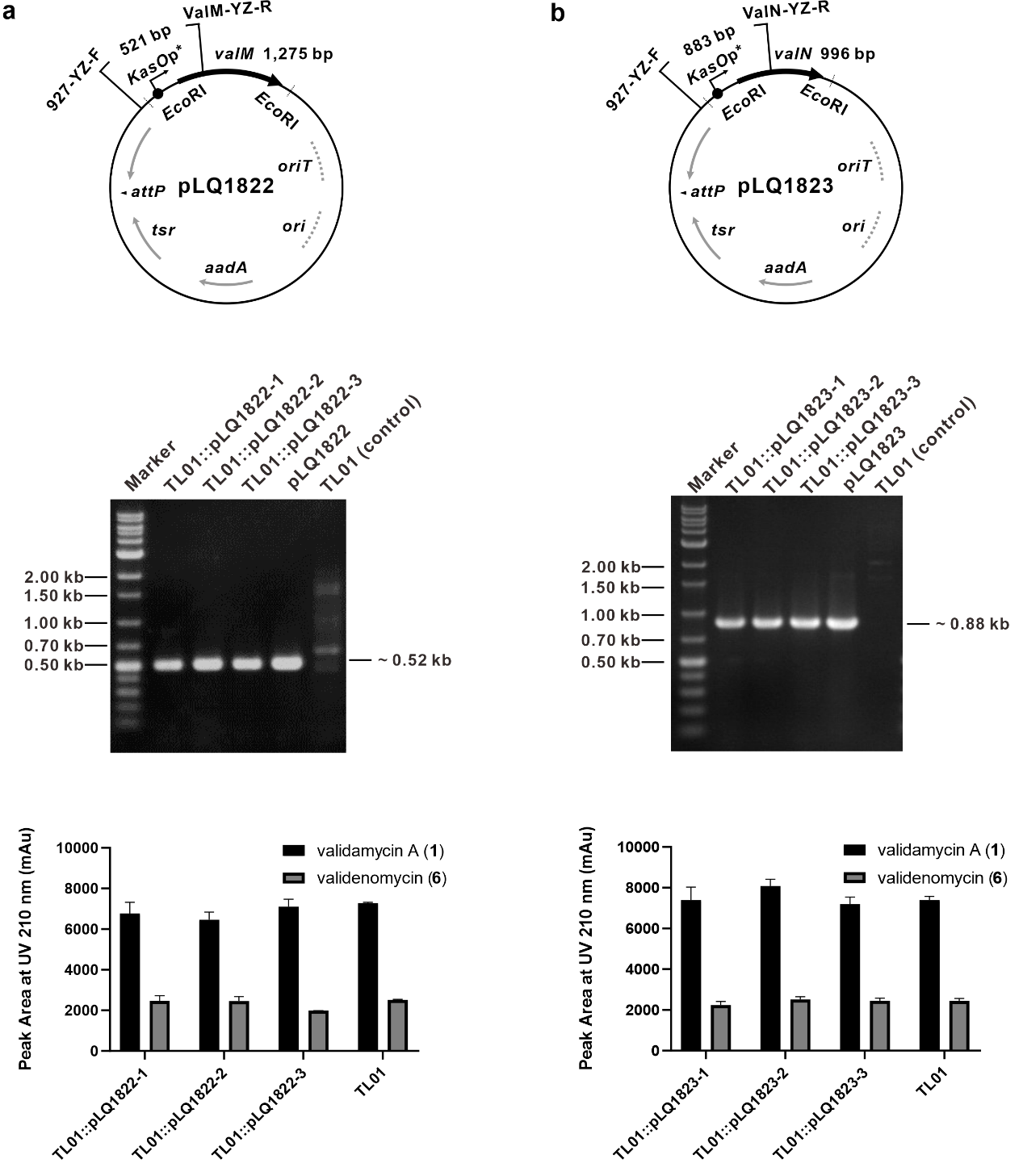


**Supplementary Figure 13. Metabolic engineering strategies for substrate supply improvement show no effect on the production of validamycin A (1) and validenomycin (6).**

**a**, Schematic map of the plasmid pLQ1822. This plasmid was constructed with pPM927 as the backbone, incorporating the gene *valM* under the control of *kasO*p*** promoter. **b**, Schematic map of the plasmid pLQ1823. This plasmid was constructed with pPM927 as the backbone, incorporating the gene *valN* under the control of *kasO*p*** promoter. **c**, Verification of TL01::pLQ1822 strain by gel electrophoresis of PCR products. PCR was performed using 927-YZ-F and ValM-YZ-R as primers. Lanes were as follows: M, DNA marker; TL01::pLQ1822-1 to TL01::pLQ1822-3, PCR product using TL01::pLQ1822-1 to TL01::pLQ1822-3 genomic DNA as template; pLQ1822, PCR product using pLQ1822 plasmid DNA as template; TL01, PCR product using TL01 genomic DNA as template. **d**, Verification of TL01::pLQ1823 strain by gel electrophoresis of PCR products. PCR was performed using 927-YZ-F and ValN-YZ-R as primers. Lanes were as follows: M, DNA marker; TL01::pLQ1823-1 to TL01::pLQ1823-3, PCR product using TL01::pLQ1823-1 to TL01::pLQ1823-3 genomic DNA as template; pLQ1823, PCR product using pLQ1823 plasmid DNA as template; TL01, PCR product using TL01 genomic DNA as template. **e**, The titers of validamycin A (**1**) and validenomycin (**3**) of TL01::pLQ1822-1, TL01::pLQ1822-2, TL01::pLQ1822-3 and TL01 after fermentation for 4 days. **f**, The titers of **1** and **3** of TL01::pLQ1823-1, TL01::pLQ1823-2, TL01::pLQ1823-3 and TL01 after fermentation for 4 days.

**Reference**

[1] Paget MSB, Chamberlin L, Atrih A, Foster SJ, and Buttner MJ. Evidence that the extracytoplasmic function sigma factor δ^E^ is required for normal cell wall structure in *Streptomyces coelicolor* A3(2)*.* Journal of Bacteriology 1999; 181(1): 204-11. <https://doi.org/10.1128/jb.181.1.204-211.1999>.

[2] Zhou X, Wu H, Li Z, Zhou X, Bai L, and Deng Z. Over-expression of UDP-glucose pyrophosphorylase increases validamycin A but decreases validoxylamine A production in *Streptomyces hygroscopicus* var. *jinggangensis* 5008*.* Metabolic Engineering 2011; 13(6): 768-76. <https://doi.org/10.1016/j.ymben.2011.10.001>.

[3] He Y. Two pHZ1358 derivative vectors for efficient gene knockout in *Streptomyces.* Journal of Microbiology and Biotechnology 2010; 20(4): 678-82. <https://doi.org/10.4014/jmb.0910.10031>.

[4] Smokvina T, Mazodier P, Boccard F, Thompson CJ, and Guérineau M. Construction of a series of pSAM2-based integrative vectors for use in *Actinomycetes.* Gene 1990; 94(1): 53-9. <https://doi.org/10.1016/0378-1119(90)90467-6>.

[5] Letunic I and Bork P. Interactive Tree of Life (iTOL) v6: recent updates to the phylogenetic tree display and annotation tool*.* Nucleic Acids Research 2024; 52(W1): W78-W82. <https://doi.org/10.1093/nar/gkae268>.
